# Molecular and Functional Characterization of Novel Fructosyltransferases and Invertases from *Agave tequilana*

**DOI:** 10.1371/journal.pone.0035878

**Published:** 2012-04-30

**Authors:** Celso Cortés-Romero, Aída Martínez-Hernández, Erika Mellado-Mojica, Mercedes G. López, June Simpson

**Affiliations:** 1 Department of Plant Genetic Engineering, Cinvestav-Irapuato, Irapuato, Guanajuato, Mexico; 2 Colegio de Postgraduados Campus Campeche, Sihochac, Champotón, Campeche, Mexico; 3 Department of Biotechnology and Biochemistry, Cinvestav-Irapuato, Irapuato, Guanajuato, Mexico; Griffith University, Australia

## Abstract

Fructans are the main storage polysaccharides found in Agave species. The synthesis of these complex carbohydrates relies on the activities of specific fructosyltransferase enzymes closely related to the hydrolytic invertases. Analysis of *Agave tequilana* transcriptome data led to the identification of ESTs encoding putative fructosyltransferases and invertases. Based on sequence alignments and structure/function relationships, two different genes were predicted to encode 1-SST and 6G-FFT type fructosyltransferases, in addition, 4 genes encoding putative cell wall invertases and 4 genes encoding putative vacuolar invertases were also identified. Probable functions for each gene, were assigned based on conserved amino acid sequences and confirmed for 2 fructosyltransferases and one invertase by analyzing the enzymatic activity of recombinant Agave protein s expressed and purified from *Pichia pastoris.* The genome organization of the fructosyltransferase/invertase genes, for which the corresponding cDNA contained the complete open reading frame, was found to be well conserved since all genes were shown to carry a 9 bp mini-exon and all showed a similar structure of 8 exons/7 introns with the exception of a cell wall invertase gene which has 7 exons and 6 introns. Fructosyltransferase genes were strongly expressed in the storage organs of the plants, especially in vegetative stages of development and to lower levels in photosynthetic tissues, in contrast to the invertase genes where higher levels of expression were observed in leaf tissues and in mature plants.

## Introduction

*Agave* species are carbohydrate rich plants that synthesize fructans as the main storage polysaccharides with starch found to be present only in low levels [1]. Non-reducing fructans are a structurally diverse group of carbohydrates consisting of fructose polymers linked to an initial sucrose moiety, and are considered to be present in approximately 15% of flowering plants [2]. Fructans in *Agave* are synthesized and stored in the structure known as the “piña", composed of stem and basal leaf tissue. Previous characterization of the fructan structures present in *Agave tequilana*, revealed a complex mixture consisting of mixed fructans (graminans) and neo-fructans, with degrees of polymerization (DP) ranging from 3 to 30 and with both β-(2–1) and β-(2–6) linkages [3], [4]. Studies of fructan content and DP in this species at different developmental stages, revealed these characteristics to be age dependent. For example, 97% of the carbohydrates present in 4 and 6.5 year old Agave plants were found to be fructans, whereas younger plants (2 years old) contained around 69% with the remaining carbohydrate content made up of fructose, glucose and sucrose. In addition the highest DPs were found in 4 year old plants [4].

Most Agave species have long life cycles and grow vegetatively for several years (usually about 5–8 for *A. tequilana*) before the emergence of the inflorescence (quiote), the sign of physiological maturity. During this long vegetative period, plants accumulate fructans as reserve carbohydrates, and although carbohydrate storage is probably the main biological function of Agave fructans, several alternative roles such as drought, cold tolerance and osmoregulation have been proposed for fructans in other plant species [5]–[7], and may also be employed in Agave species which are well adapted to arid and semiarid regions, where environmental conditions change drastically in terms of precipitation and temperature [8].

Several fructan structures have previously been characterized in other plant species and differ by their length, DP, branching, linkage between adjacent fructose molecules and the position of the glucose residue [9]. Inulins or linear type fructans (β-(2–1) linkages between fructose residues) are usually found in plants belonging to the order Asterales, whereas the linear levan type with β-(2–6) linkages between fructose residues are found in some grasses such as *Dactylis glomerata*. Graminan type fructans with both β-(2–1) and β-(2–6) linkages are present in certain cereals such as wheat and barley [10]. A recent study however also reported the presence of graminan type fructans in *Pachysandra terminalis,* a dicotyledonous species [11]. The complex neo-fructans contain both β-(2–1) and β-(2–6) linkages, with fructan moieties linked to both the fructose and glucose residues of the basic sucrose molecule and have been obtained from Asparagales species such as *Allium cepa* [12], *Asparagus officinalis* [13] and the Poaceae species *Lolium perenne* [9]. In many cases a single fructan class is found in a particular species, however in some species a heterogeneous mixture of different fructan classes with different DPs and branching structures is found as shown for *A. officinalis* and *A. cepa* [13] and for *A. tequilana* var. *azul* [3], [4].

Fructan biosynthesis is carried out by fructosyltransferases (FTs) and based on the fructan structures previously described for *A. tequilana* var. *azul* [14], at least three different enzymes are required for their synthesis: Sucrose:sucrose1-fructosyltransferase (1-SST), fructan: fructan 6G-fructosyltransferase (6G-FFT) and sucrose: fructose 6-fructosyltransferase (6-SFT). In contrast, 1 and 6 fructan exohydrolases (FEHs) are responsible for releasing terminal β-D fructofuranose residues from the different forms of fructo-oligosaccharides [15]–[21]. Genes encoding FTs and FEHs from several plant species have previously been isolated and functionally characterized (for a review see [22]) including a gene encoding a Sucrose:sucrose 1-fructosyltransferase (1-SST: DQ535031) from *A. tequilana* [23].

Plant FTs belong to family 32 of the glycoside hydrolases (http://afmb.cnrs-mrs.fr/CAZY). This family also includes the acid invertase enzymes found in the vacuole or apoplast [24] but not the neutral invertases which are classified as members of family GH100 and are found in the cytosol and in plant organelles [25]. Invertases are responsible for the hydrolysis of sucrose to glucose and fructose and unlike the fructosyltransferases these enzymes are present in all plant species. Invertases have also been associated with important physiological processes such as tissue growth and the storage of carbohydrates in sink organs and their metabolism in source organs [26]. Plant FTs, FEHs and invertases show many similarities and phylogenetic analyses suggest that FTs evolved from vacuolar invertases whereas FEHs are more closely related to cell wall invertases [27].

In contrast to cDNA sequences, fewer genomic sequences encoding fructosyltransferases and invertases have been described. A conserved exon/intron structure for some fructosyltransferase and invertase genes including in several species the presence of a 9 bp mini-exon encoding 3 amino acids (DPN) which constitute part of the WMNDPNG motif which determines substrate specificity has been reported. The mini-exon was shown to undergo alternative splicing under cold stress in potato [28] and a splice variant at a different exon-intron junction has also been observed in cotton [29].

This report describes the identification and characterization of cDNAs encoding two types of fructosyltransferases (1-SST and 6G-FFT) and two types of invertases (cell-wall and vacuolar) from *Agave tequilana*. Comparisons with genomic sequences were carried out for all identified genes and selected genes were chosen to analyze enzyme activity in a heterologous expression system (*Pichia pastoris*) and/or study gene expression patterns by qRT-PCR.

## Results

### Identification of Agave Fructosyltransferase and Invertase ESTs

**Figure 1:**
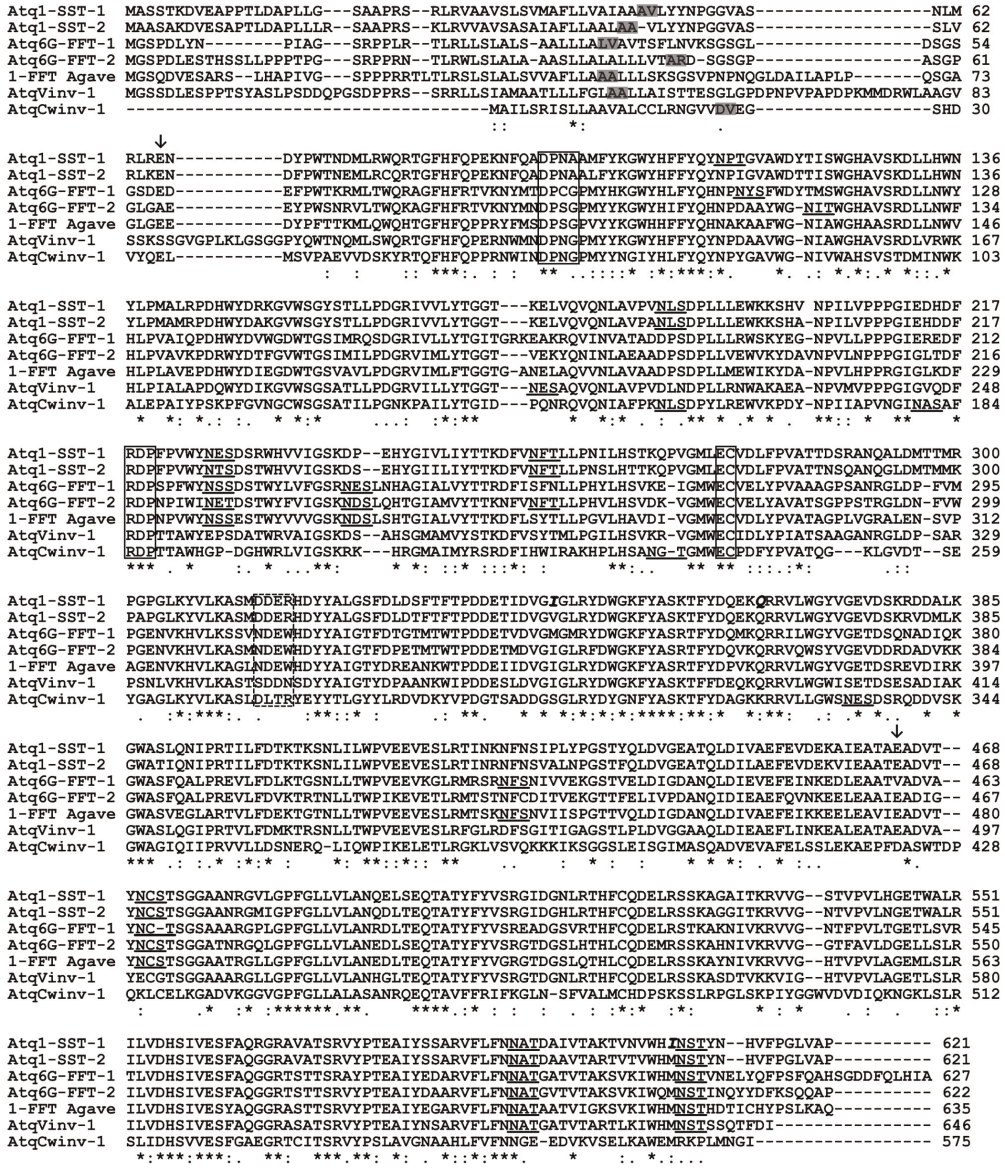
Alignment of deduced amino acid sequences of fructosyltransferases and Invertases of *A. tequilana*. Asterisks, colons and periods indicate identical residues, conserved substitutions, and semi-conserved substitutions, respectively. Putative initiation points of the large and small subunits are arrowed. Potential glycosylation sites are underlined. The β-fructosidase motif, RDP motif and the cysteine catalytic site are boxed. A sucrose-donor substrate motif is shown as a stippled box. Predicted leader sequence cleavage points are shaded in grey. Differences between *Atq1-SST-1* and SSTAg are shown in italics.

A BLAST search carried out within an *A. tequilana* transcriptome database obtained by the sequencing of around 29,000 clones of cDNA libraries (Martínez-Hernández et al. in preparation) revealed 33 sequences with highly significant homology to fructosyltransferase (FT) or invertase genes. According to the annotation of these sequences, 13 ESTs showed highest homology to 1-SST, 8 to 6G-FFT, 7 to vacuolar invertases and 5 to cell wall invertases. The ESTs obtained ranged in size from 532–1224 bp and comparison of the nucleotide and translated amino acid sequences of each EST, suggested that two distinct genes encoding 1-SST and two distinct genes encoding 6G-FFT had been identified. These putative fructosyltransferase encoding genes were named *Atq1-SST-1* and *-2* and *Atq6G-FFT-1* and *-2* respectively. Comparison of the ESTs with previously annotated sequences of invertases, allowed the identification of 4 different cell wall invertase genes and 4 different vacuolar invertase genes. Clones with complete ORFs for all four of the fructosyltransferase genes were identified, whereas clones carrying the complete ORF of only 1 cell wall invertase (denominated *AtqCwinv-1*) and one vacuolar invertase gene (denominated *AtqVinv-1*) were identified among our cDNA collection. Clones carrying the complete ORF of each gene were re-sequenced and confirmed sequences were deposited in GenBank under accession numbers JN790053-JN790058. *In silico* translation of the ORFs allowed the determination of the complete, putative amino acid sequence corresponding to all of the identified genes. Alignment of the amino acid sequences of the two *A. tequilana* fructosyltransferase proteins with sequences available in GenBank (1-FFT EU026119 and 1-SST DQ535031) and the complete amino acid sequences of the six putative fructosyltransferase/invertase proteins identified in this work shows the degree of conservation at the sequence level and the features characteristic of glycoside hydrolase family 32 (Figure 1). Conserved residues within the catalytic large subunit domain are boxed and whereas the RDP and cysteine catalytic motifs (EC) are identical for all proteins, the WMNDPNG motif varies with respect to the putative function of the protein. The vacuolar and cell wall invertases have the general consensus sequence DPNG whereas the 1-SST proteins have DPNA, Atq6G-FFT2 and the putative 1-FFT protein (Acc. EU026119) have DPSG and Atq6GFFT-1 has DPCG. A stippled box indicates a second motif, shown to be important for the determination of sucrose as donor substrate. Atq1SST-1, Atq1SST-2 and AtqCwinv1 all maintain the Asp/Arg (D/R) couple thought necessary for determination of sucrose as a donor substrate whereas Atq6G-FFT-1 and Atq6G-1FFT-2, which utilize primarily 1-kestose as a substrate have an Asn/Trp couple (N/W). Surprisingly AtqVinv-1 has a very different motif Ser/Asn (S/N) at this position suggesting that sucrose may not be the donor substrate in this case. Arrows indicate the putative cleavage points where the initial protein is processed to produce 2 subunit polypeptides and the deduced signal peptide cleavage points are shaded in grey.

The sequence obtained for Atq1-SST-1 differed by 18 nucleotides in the ORF from the sequence reported by Avila-Fernández et al. [23] denominated SSTAg (DQ535031). These nucleotide differences were mainly neutral (15/18) and only 3 amino acid changes (I to V position 344, Q to H position 365 and I to M position 607, Figure 1) were observed between Atq1-SST-1 and SSTAg (99.5% identity), suggesting that *Atq1-SST-1* and *SSTAg* are probably distinct alleles of the same gene. *Atq1-SST-2* however shows significant differences both at the nucleotide level (87% of identity), and at the amino acid level (89% identity) with *Atq1-SST-1* (Figure 1), suggesting that this sequence represents yet another allele or possibly a second *A. tequilana 1-SST* gene. In contrast, *Atq6G-FFT-1* and *Atq6G-FFT-2* clearly encode distinct proteins showing lower levels of identity than the Agave 1-SST genes, with 75% identity at the nucleotide level and 70% at the amino acid level (Figure 1). A summary of the deduced characteristics of the sequenced genes and translated proteins is presented in Table 1.

**Figure 2:**
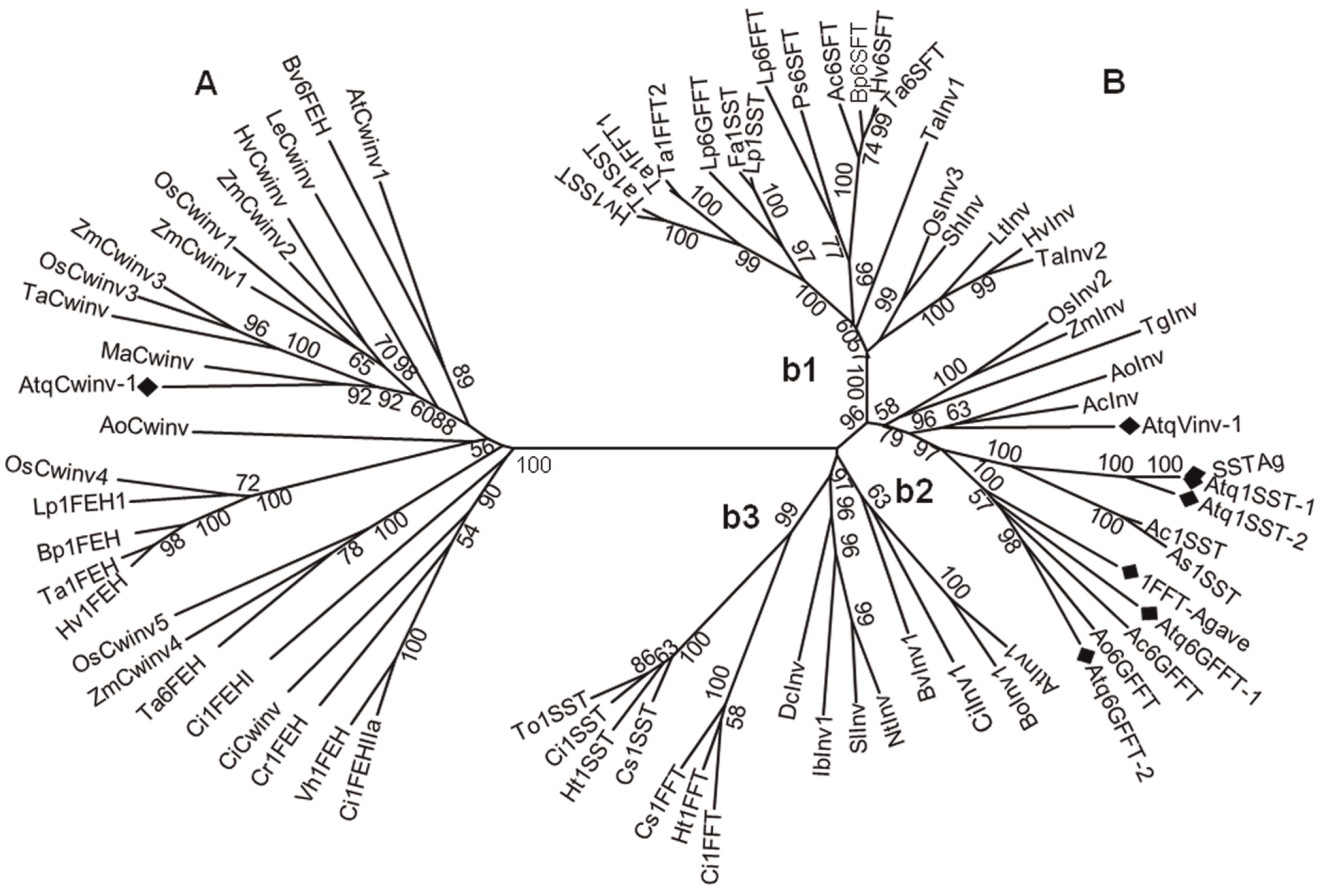
Unrooted tree of selected fructosyltransferases, fructan exohydrolases and invertases from monocotyledonous and dicotyledonous plants. A: branch containing cell wall invertases and FEHs, B: branch containing vacuolar invertases and fructosyltransferases, b1/b2-monocotyledons, b3-dicotyledons. *A. tequilana* fructosyltransferases and invertases are indicated with a diamond. Accession numbers are provided in Table S1.

**Table 1:** Molecular features of fructosyltransferases and invertases from *A. tequilana*.

Gene	No. ESTs	cDNA length(pb)	Exon/IntronLength(pb)	No. Aminoacids	SignalPeptide (aa)	Glycosilation Sites(NXT/S)	MolecularMass (Kd)	Iso-electricpoint
*Atq1-SST-1*	11	2164	4050	621	48	7	69.8	5.76
*Atq1-SST-2*	2	2176	3645	621	47	6	69.2	5.76
*Atq6G-FFT-1*	6	2087	4289	627	34	7	70.7	5.57
*Atq6G-FFT-2*	2	2078	5220	622	50	7	69.7	5.11
*AtqVinv-1*	3	2564	3643	646	48	3	70.4	5.4
*AtqCwinv-1*	1	1872	4636	575	24	4	64.2	8.38

Comparison of the amino acid sequences obtained from the cDNAs carrying the complete ORF of the fructosyltransferases and invertases from Agave with those from other plant species produced the dendrogram shown in Figure 2. Accession numbers for all sequences used in the analysis can be found in Table S1. Two main groups were obtained, one (A) containing sequences classified as cell wall invertases and fructan exohydrolases and the other (B) containing sequences classified as vacuolar invertases and fructosyltransferases. Many of the proteins included in Figure 2 have been functionally characterized (a non-exhaustive list is included in Table S2), supporting the overall classification for each group. Additionally the difference in isoelectric points (Table 1) and analysis of the N-terminal regions using the SignalP program (data not shown), all support a distinct classification for AtqCwinv and AtqVinv.

Within each large group, clusters containing proteins for specific enzymes and for either monocotyledons (b1/b2) or dicotyledons (b3) can be observed with Bootstrap values supporting the most important branches. The most closely related fructosyltransferase sequences from other species are those encoding 1-SST enzymes from *A. cepa* and *A. sativum,* 70 and 68% of identity respectively to Atq1-SST-1 and 69% and 66% identity respectively to Atq1-SST-2 at the amino acid level. On the other hand, the genes most closely related to the *A. tequilana* 6G-FFT genes are those from *A. cepa* and *A. officinalis.* Atq6G-FFT-1 shows around 63% of identity to the most closely related *A. cepa* sequence and 67% to that of *A. officinalis.* Atq6G-FFT-2 shows even higher identities to these genes, around 70% to *A. cepa* and 74% to *A. officinalis.*

The putative vacuolar invertase from *A. tequilana* also shows closest homology with other members of the order Asparagales with around 73% of identity to a vacuolar invertase from *A. officinalis* and a lower percentage to that of *A. cepa* (67%).

In contrast to the other genes *AtqCwinv-1*, a putative cell wall invertase, is most closely related (69% identity), to a similar protein from *Musa acuminata* (a member of the order Zingiberales) with lower values of identity observed with respect to cell wall invertases from *Oryza sativa* (60%) and *Hordeum vulgare* (56%) both members of the Poaceae.

### Functional Characterization of Recombinant Atq6G-FFT-1, Atq1-SST-2 and AtqCwinv-1 in *Pichia pastoris*

Based on the novelty of the *Atq1SST-2* sequence in comparison to *Atq1SST-1* and the differential pattern of expression observed for *Atq6G-FFT1,* these genes were chosen for functional analysis in addition to *AtqCwinv-1* in order to determine whether the protein encoded by the latter gene showed fructan exohydrolase activity.

The cDNAs encoding the putative mature protein of each chosen gene were cloned into the pPICZαA vector, in frame with the α-factor signal for extracellular secretion and behind the 6×His tag. Recombinant proteins were visualized on a 10% SDS-PAGE stained with Coomassie brilliant blue and positively identified by Western blot using an anti-His antibody. Products were observed in the expected size range from around 75 kD for Atq1-SST-2 and AtqCwinv-1 to 80 kD for Atq6G-FFT-1 (data not shown).

**Figure 3:**
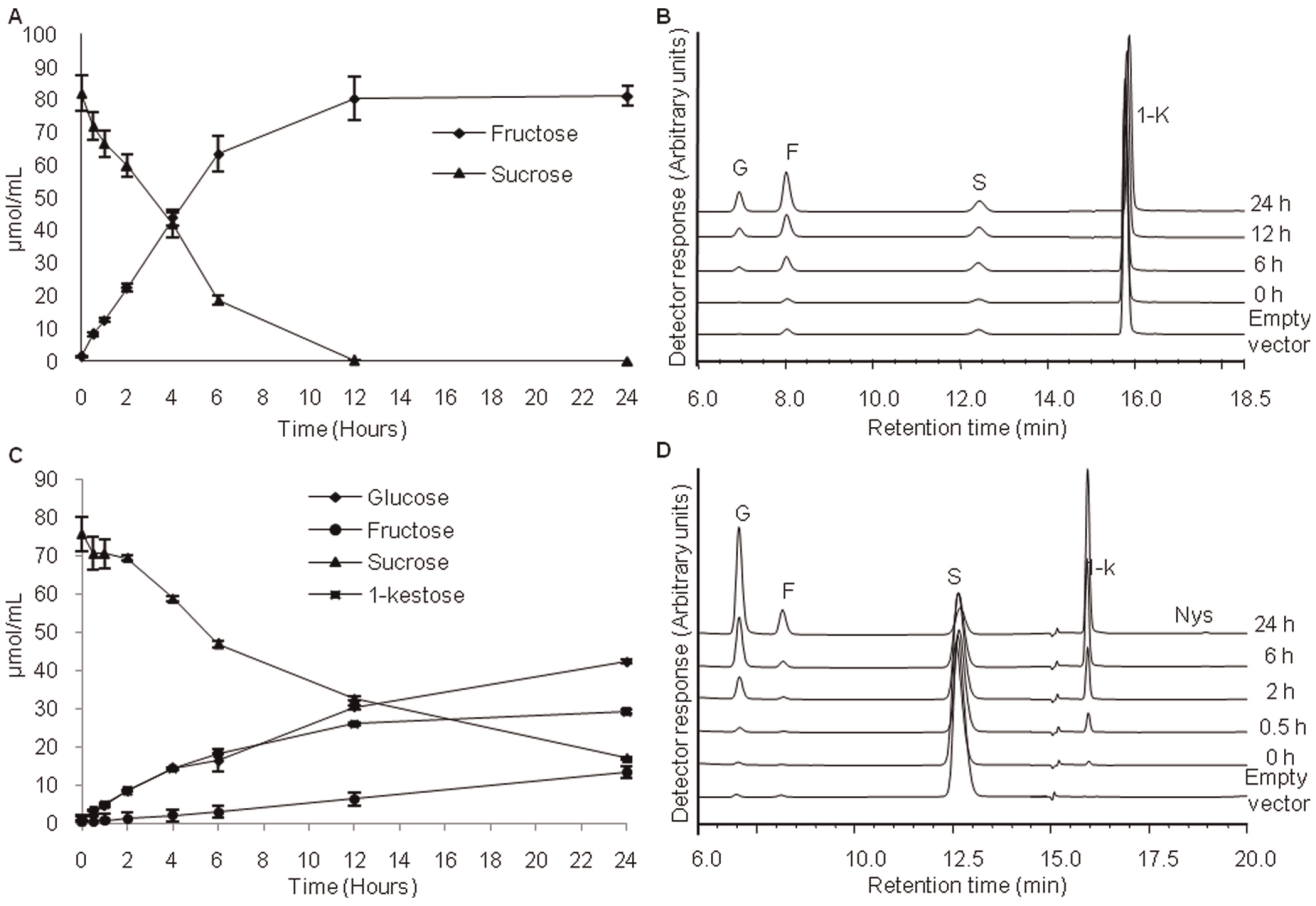
Activities of recombinant AtqCwinv-1 and Atq1-SST-2 proteins. A. Time-course of reaction products when 100 mM sucrose is supplied as substrate for AtqCinv-1. B. HPAEC chromatograms of the products when 100 mM 1-kestose was supplied as substrate for AtqCinv-1. C. Time course of reaction products of Atq1-SST2 when 100 mM sucrose is supplied as substrate and D. HPAEC chromatograms of the products of the Atq1-SST2 reactions when 100 mM sucrose was supplied as substrate. G-glucose, F-fructose, S-sucrose, 1-K-1-kestose, Neo-Neokestose, Nys-nystose.

Enzymatic reactions were carried out by incubating the purified recombinant proteins with sucrose, 1-kestose or both, at 30°C for different time intervals. The products of each reaction were identified by thin layer chromatography (TLC) and it was observed that AtqCwinv-1 produced fructose and glucose when incubated with sucrose as the sole substrate (data not shown), supporting the previously predicted hydrolyzing activity. Possible exohydrolase activity could not however be clearly established by TLC. Results of HPAEC-PAD analysis confirmed the invertase activity shown by TLC, with complete hydrolysis of sucrose occurring after 12 h of incubation (Figure 3A), and also showed that some exohydrolytic activity occurred when sucrose was replaced by 1-kestose as substrate, suggesting that AtqCwinv-1 has both invertase and fructan exohydrolase activity (Figure 3B).

When protein extracts of the *P. pastoris* strain carrying the *Atq1-SST-2* gene were incubated with 100 mM sucrose as substrate, 1-kestose and glucose were the main products obtained indicating the transfer of a fructosyl residue from one sucrose molecule to another (Figure 3C) and confirming the identity of this gene as a 1-SST type fructosyltransferase. After 24 h of incubation, around 78% of the sucrose substrate was consumed. Interestingly, a small amount of nystose a higher degree fructan polymer is also observed (Figure 3D).

**Figure 4:**
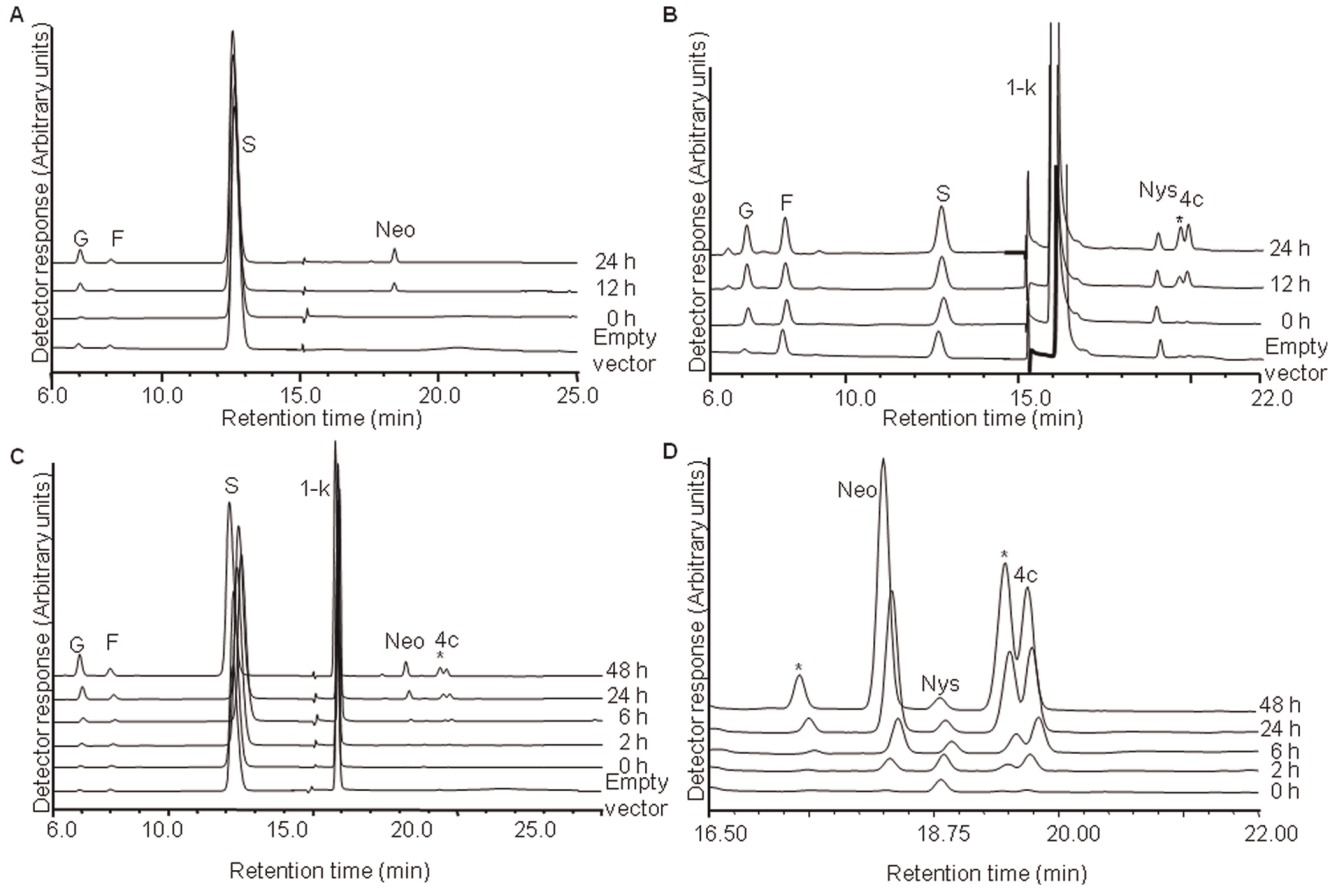
Activity of recombinant 6G-FFT-1 protein. HPAEC chromatograms of the products obtained by the reactions supplied with: A. 100 mM sucrose. B. 50 mM 1-kestose C. both 100 mM sucrose and 50 mM 1-kestose D-amplification of the chromatogram in C. Abbreviations G-glucose, F-fructose, S-sucrose, 1-K-1-kestose, Neo-Neokestose, Nys-nystose, 4c-1^F^,6^G^-Di-β-D-fructofuranosylsucrose. Unidentified products are indicated by asterisks.

The predicted activity of the Atq6G-FFT-1 enzyme is the production of the trisaccharide neokestose and both 1-kestose and sucrose are necessary components of the reaction mixture. When the recombinant protein was incubated with 100 mM sucrose, neokestose was observed after 12 h incubation but no products of a higher degree of polymerization were observed (Figure 4A). When 1-kestose was used as substrate no neokestose was produced but a tetrasaccharide 4c molecule was generated (Figure 4B).

When both 100 mM of sucrose and 50 mM of 1-kestose were included in the reaction both neokestose and the 4c tetrasaccharide moiety were observed (Figure 4C and D). Comparison with standards determined the 4c molecule to be a tetrasaccharide with an FGFF type structure (1^F^,6^G^-Di-β-D-Fructofuranosylsucrose) [30]. Trace amounts of nystose were found as an impurity in the 1-kestose stock, however the nystose peak does not change in size indicating that it is not used either as an acceptor or donor for fructan polymerization. Two additional peaks, one with a retention time of 17.51 min and the other closely associated with the 4c molecule at 19.53 min (indicated with * in Figure 4, B, C and D) were observed at incubation times of 12 h and longer however it was not possible to identify these products with the available standards.

### Genomic Structure of Agave Fructosyltransferases and Invertases

**Figure 5:**
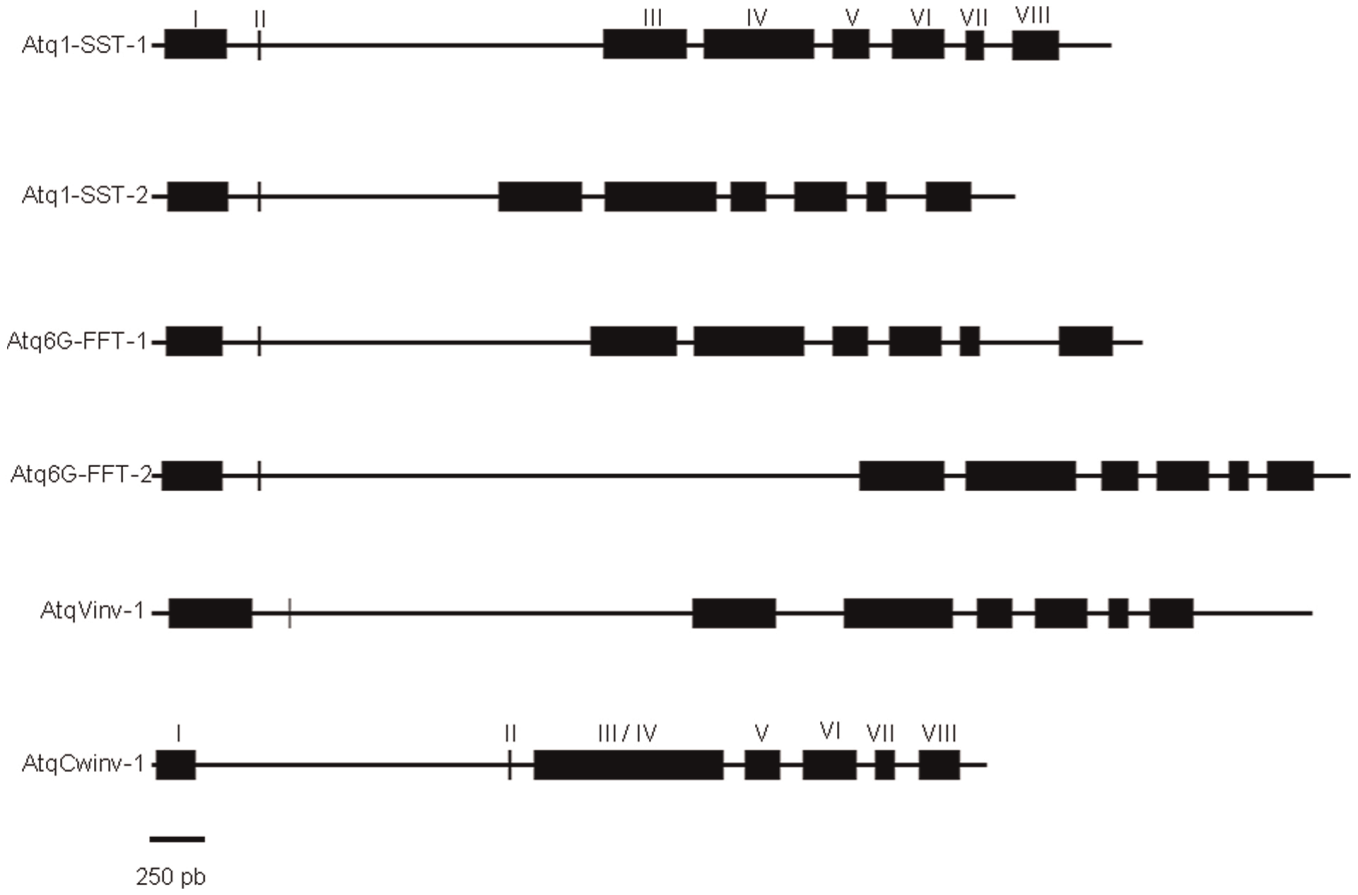
Schematic representation of the genomic structures of *A. tequilana* Fructosyltransferases and Invertases. Introns are represented by lines and exons with solid boxes. Exons are distinguished by Roman numerals from left to right. Only *AtqCwinv-1* shows a distinct pattern of exon/intron number and organization. Drawings are to scale and scale bar represents 250 bp.

The Agave genes for which cDNAs with complete ORFs were available were characterized at the genomic level by designing specific PCR primers spanning intron regions (sequences are deposited in GenBank under Accesion numbers JN790059-JN790064). Comparison between the cDNA and genomic sequences revealed a conserved exon/intron structure of 8 exons and 7 introns for all genes with the exception of *AtqCwinv-1* comprised of 7 exons and 6 introns. All genes carry a nine base pair mini-exon and in general the sizes and spacing of introns/exons were conserved (Figure 5). The length of intron 2 was found to be most variable ranging in length from 1081 bp in *Atq1-SST-2* to 2716 bp in *Atq6G-FFT-2*. Exons 3 and 4 of *AtqCwinv-1* are apparently fused in comparison to the other genes and some other mutational event in introns 1 or 2 may have occurred causing the change in position of the mini-exon closer to exon 3 without altering the position of the characteristic Sucrose Binding Box found in the 9 bp mini-exon or FRD and EC motifs within the coding sequence. Most of the splice sites are denoted by the typical GT-AG motifs, however the splice site between exon 6 and 7 of the FTs and *AtqVinv-1* and between exon 4 and 5 of *AtqCwinv-1* present a GC-AG motif (data not shown).

### Analysis of Expression of Agave Fructosyltransferases and Invertases

Semi-quantitative RT-PCR analysis was carried out in different tissues and at different developmental stages. Samples were obtained from stem (S), base of leaf (BL) and mid-leaf (ML) as shown in Figure S1, from plants of 1, 3 (pre-flowering, vegetative stage), 5 and 7 (post-flowering, mature stage) years of age. All four fructosyltransferase genes were expressed in all tissues and at all developmental stages. However *Atq6G-FFT-1* showed different levels of expression whereas *Atq6G-FFT-2* was constitutively expressed. In contrast *Atq1-SST-1* and *Atq1-SST-2* showed similar differential patterns and levels of expression (Figure S2).

**Figure 6:**
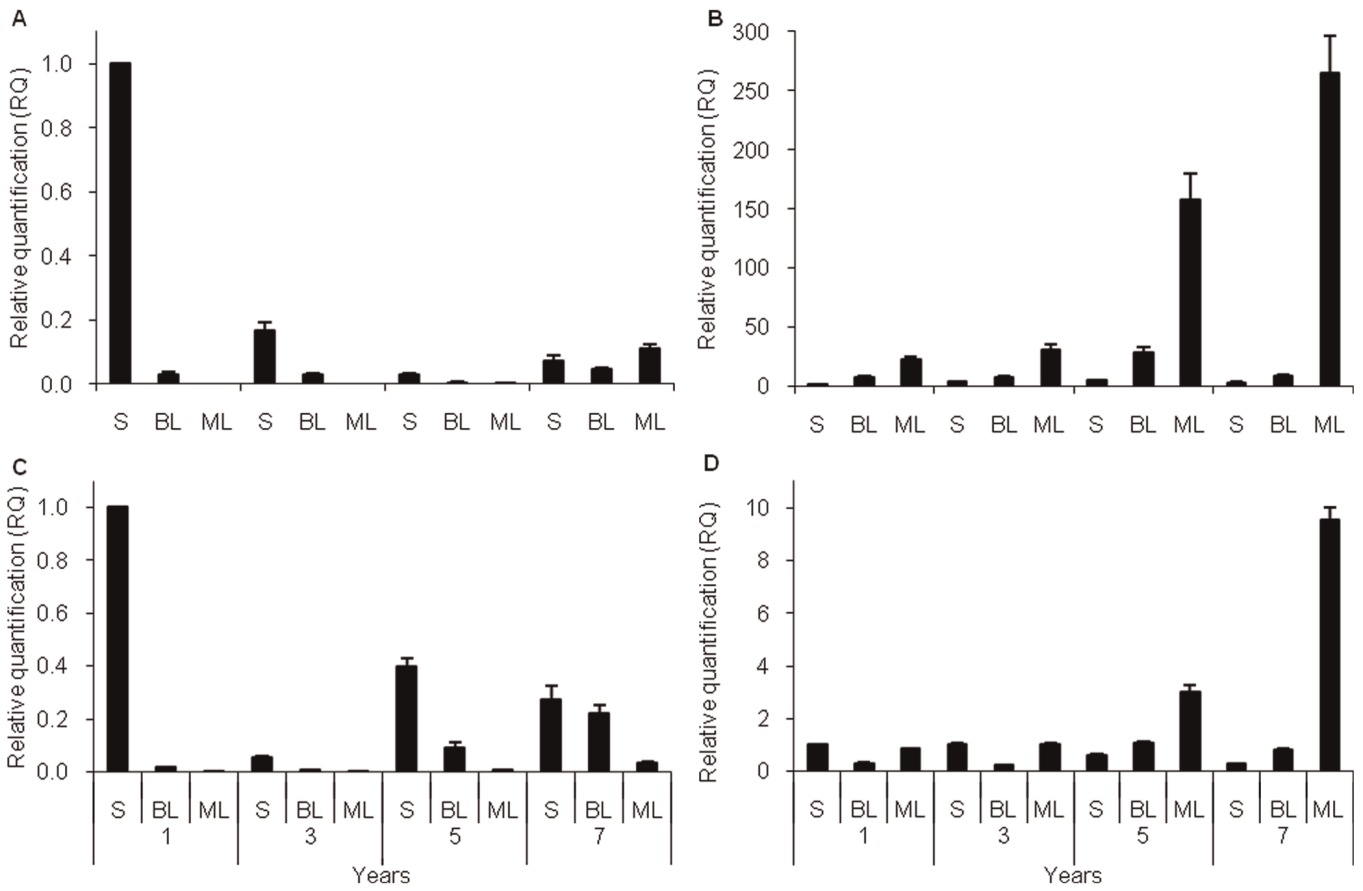
qRT-PCR expression profiles of *A. tequilana* fructosyltransferase and invertase genes in different plant tissues and developmental stages. A. *Atq1-SST-2,* B. *AtqVinv-1*, C. *Atq6G-FFT-1*, D. *AtqCwinv-1.* S-stem, BL-Base of leaf and ML-Mid-leaf of 1 and 3, year old plants (vegetative stage) and 5 and 7 year old plants (Post-reproductive stage). Photographic examples of tissues sampled are shown in Figure S1.

Based on these results, *Atq1SST-2*, *Atq6G-FFT-1* and both invertase genes were chosen to carry out qRT-PCR, in order to obtain more precise data on the expression levels and patterns of each gene. In the samples from 1 and 3 year old plants, *Atq1-SST-2* and *Atq6G-FFT-1* showed very similar expression patterns, with the highest level of expression in stem tissue of one year old plants, much lower expression in BL and very low levels of expression in ML tissue (Figure 6A and C). This pattern of expression was similar in 3 year old plants for both genes, although the level of expression in stems of 3 year old plants was around 5 to 10 × lower in comparison to expression in 1 year old stems. Differences in expression patterns between *Atq1-SST-2* and *Atq6G-FFT-1* were observed in 5 and 7 year old plants. Even lower levels of expression were observed for *Atq1-SST-2* in stem tissue from 5 year old plants in comparison to 3 year old samples and this was also observed in BL and ML tissue. In 7 year old plants *Atq1-SST-2* was more highly expressed in all tissues than in the 5 year old samples with highest expression in ML tissue in contrast to the samples for this tissue from 1, 3 and 5 year old plants. *Atq6G-FFT-1* showed the same pattern of decreased expression in BL and ML tissue for 5 and 7 year old samples however levels of expression in stem tissue were higher than in 3 year old plants. Levels of expression in BL and ML were also found to be higher than both 3 and 1 year old plants (Figure 6C).

In contrast to the fructosyltransferase genes, the invertase genes showed highest levels of transcript accumulation in photosynthetic tissues, especially in mature plants. *AtqVinv-1* showed the highest relative increase in expression levels of all the genes analyzed and a consistent pattern of expression increasing from stem to leaf tissues can be observed for samples from each year with strong expression in ML sections in mature plants (5 and 7 years) (Figure 6B). On the other hand, *AtqCwinv-1* shows much lower levels of change in expression in comparison to *AtqVinv-1*. In years 1 and 3 highest levels of expression were observed in stem and ML tissue whereas in years 5 and 7 a relative increase was observed in BL tissue and a significant increase in ML tissue with the highest level of expression in 7 year old ML tissue (Figure 6D).

## Discussion

The fructosyltransferase/invertase genes identified were found to group according to their putative activities and to the most closely related plant species such as *A. cepa* and *A. officinalis* when their deduced amino acid sequences were compared with those of other plants. The close association between the vacuolar invertase and the fructosyltransferase genes and the much weaker association with the cell wall invertase genes supports the hypothesis that FTs evolved from vacuolar invertases whereas cell wall invertases are more closely related to fructan exohydrolases [27], [31]. The identification of several distinct vacuolar and cell wall invertase genes in *A. tequilana* also agrees with previous reports for several other species including rice, poplar and *A. thaliana* [32]–[35].

Surprisingly, no ESTs encoding either a 6-SFT enzyme or showing close homology to the Agave 1-FFT sequence available in GenBank were identified suggesting that transcripts encoding these enzymes are expressed at very low levels. Alternatively the Agave 6G-FFT enzymes may carry out additional 6-SFT or 1-FFT activities. Dual activity has previously been reported for 6G-FFT/1-FFT enzymes from *Asparagus* [34], *Allium* [12] and *Lolium* [9] where in addition to the formation of neokestose, products with β-(2–1) linkages are also synthesized. Despite the close homology between *Atq6G-FFT-1* and 6G-FFT from *A. officinalis*, the results obtained for recombinant Aqt6G-FFT-1 *in vitro* do not show evidence of dual activity, however this cannot be ruled out since such activity may depend on substrate concentration or other experimental conditions.

Two distinct genes were identified for both 1-SST and 6G-FFT enzymes. *Atq1-SST-1* corresponds to the gene denominated SSTAg described by [23] whereas based on nucleotide and amino acid differences Atq1-SST-2 is a distinct gene or allele. Interestingly ESTs for both genes were identified in the cDNA library obtained from ovarian tissue with Atq1-SST-2 sequences found exclusively in this tissue whereas ESTs for Atq1-SST-1 were also found in libraries from inflorescence/apical meristems, leaf and bulbil tissue [36]. Six ESTs were identified for Atq6G-FFT-1 in cDNA libraries from floral and bulbil tissues, whereas 2 ESTs for Atq6G-FFT-2 were identified in libraries from stem and floral tissues. The observed expression pattern suggests a possible role for fructans in reproductive tissues of Agave, perhaps in a similar manner to that reported for wheat where fructan metabolism and FT expression in anther and ovarian tissue was shown to play a role in drought tolerance in this species [37].

Conserved motifs shown in previous reports to be important for the determination of the activity of each enzyme were also identified in the deduced protein sequences from *A. tequilana* [21], [27], [38]–[40]. Specific changes within the WMNDPNG motif (DPNG) correlate well with the proposed activity of the enzyme: DPNA in Atq1-SST2, DPCG/DPSG in Atq6G-FFT-1 and -2 respectively and the conservation of the DPNG motif in both forms of invertase as described previously for other species [38], [41]. A second motif conserved in FT enzymes also correlates well with utilization of sucrose as a donor substrate. Atq1-SST-1 and Atq1-SST-2 carry the motif DDER and AtqCwinv-1 has DLTR. The Aspartic acid/arginine (D/R) combination of the flanking amino acids in this motif was shown to be important for utilization of sucrose as a donor substrate [39], [42]. In contrast Atq6G-FFT-1 and 2, which use 1-kestose as a substrate, carry NDEW at this position. This motif was shown to be important in determining enzyme specificity of a 6G-FFT enzyme from *L. perenne* [39].

AtqVinv-1 in contrast shows a non-typical motif: SDDN, even though sucrose should be the preferred substrate for this enzyme. The unexpected amino acid sequence cannot be put down to a sequencing error since the nucleotide sequence spanning these amino acids was obtained from 2 independent amplifications from genomic DNA and also the sequences of 3 independent cDNA clones. However of the first 100 hits by BLAST search of the GenBank database using the AtqVinv-1 amino acid sequence as a query, only one (a sequence annotated as a 6-SFT precursor enzyme from *P. terminalis* and previously reported by Van den Ende et al. [11] did not show the consensus motifs DXXR or DXXK at this position. The conserved amino acids in the motif have been shown to be extremely important for sucrose binding at the active site of invertases and FTs and it remains to be determined whether activity of AtqVinv-1 is affected by this modified motif.

Recombinant proteins encoded by *Atq1-SST-2*, *Atq6GFFT-1* and *AtqCwinv-1* were expressed and purified in *P. pastoris.* Although recombinant proteins were purified by using the histidine tag, no apparent interference to the binding site or protein structure was observed when modeled proteins were compared with and without the tag present (Figure S3). Avila-Fernández et al. [23] previously reported the characterization of a 1-SST cDNA (SSAg) from *A. tequilana,* which was also cloned and sequenced in the present work. Sequence analysis also identified *Atq1-SST-2* as a second gene putatively encoding this enzyme and HPAEC analysis confirmed the activity of the recombinant protein *in vitro*. Similar to SSAg [23] a small amount of nystose was produced at long reaction times indicating a low affinity of the recombinant Atq1-SST-2 enzyme for 1-kestose in relation to that for sucrose.

HPAEC analysis of the activity of the recombinant Atq6G-FFT-1 enzyme confirmed the typical activity of this class of fructosyltransferase with the production of neokestose when both sucrose and 1-kestose were supplied as substrates. Atq6G-FFT-1 also produced neokestose in the presence of sucrose as the sole substrate, in contrast to a report for *A. officinalis* where a recombinant 6G-FFT with sucrose as substrate did not produce any product [34]. However incubation of a 6G-FFT from *L. perenne* with sucrose as sole substrate produced 1-kestose and neokestose suggesting that 1-kestose is first produced from sucrose and then used as a fructose donor to produce neokestose by the *L. perenne* 6G-FFT [9]. In the case of Atq6G-FFT-1 no detectable 1-kestose was observed indicating that either production of this molecule is transient and it is immediately utilized to produce neokestose or that Atq6G-FFT-1 directly utilizes sucrose as the fructose donor to produce neokestose.

On the other hand, no formation of neokestose was observed when 1-kestose was supplied as substrate, but a tetrasaccharide (4c molecule) and an unidentified product were observed after 12 hours of incubation. It has been proposed that the 4c molecule can be formed from two molecules of 1-kestose with liberation of sucrose [34] and this could explain the formation of the observed 4c molecule at least under the experimental conditions used. However since the 1-kestose preparation used in the reaction contained a small amount of sucrose and as described above Atq6G-FFT-1 can probably utilize sucrose as a fructose donor, an alternative explanation is the incorporation of a fructose moiety from the sucrose present in the reaction mixture to directly produce the 4c molecule by addition of fructose to 1-kestose at C6 of the glucose residue.

When sucrose and 1-kestose were provided together as substrates, the typical activity for a 6G-FFT was observed with the formation of neokestose and 4c structures, confirming that Atq6G-FFT-1 is capable of carrying out these reactions at least under *in vitro* conditions. However as described above, it is possible that Atq6G-FFT-1 carries out these reactions independently and that neokestose is not a necessary intermediate for the production of the 4c tetrasaccharide.

Analysis by HPAEC-PAD demonstrated that AtqCwinv-1 was capable of carrying out both invertase and exohydrolase activities *in vitro.* Enzymes with both invertase and exohydrolase activity have been previously reported in other species including *A. thaliana* [43] and rice [44]. In such non-fructan producing species these enzymes are thought to play a role in defense by degrading fructans putatively produced by pathogens [43].

Comparison of the cDNA and genomic sequences allowed the determination of gene structure, including the size and number of exons/introns. The conserved structure between the Agave fructosyltransferase genes and the vacuolar invertase supports their shared evolution and separation from the cell wall type invertases and fructan exohydrolases since *AtqCwinv-1* shows a distinct exon/intron structure. The strong conservation at the genome level for the *A. tequilana* FTs and vacuolar invertase contrasts with other plant species where extensive variation in the pattern of introns and exons can be observed. Francki et al. [45] compared the exon/intron structure of invertases and fructosyltransferases in rice, wheat and *L. perenne* where the number of exons for these genes ranged from 2–7 in rice, 3–4 in *L. perenne* and 4–8 in wheat. Strikingly *AtqCwinv-1* shows an exon/intron pattern similar to one of the rice invertase genes whereas the other Agave genes show the same exon/intron pattern as the 8 exon wheat gene. The authors suggest that the reduction in exon number between rice and *L. perenne* and rice and wheat is due to exon fusion whereas the 8 exon wheat gene arose from the insertion of an extra exon. A similar situation may have occurred in *A. tequilana* where fusion of exons III and IV led to the *AtqCwinv-1* structure. This may indicate in evolutionary terms that the vacuolar invertase/FT encoding genes identified in *A. tequilana* diverged relatively recently and still retain many common features. The strongly conserved genome structure of the *A. tequilana* vacuolar invertase/FT genes in comparison to the rice invertases may indicate the importance of this structure for the activity of the encoded enzymes.

An EST encoding a *6G-FFT1* lacking the sequence corresponding to the 9 bp mini-exon was identified in the Agave cDNA library obtained from anthers (Figure S4), suggesting that alternative processing may occur in *A. tequilana* under specific conditions, as was shown for potato invertase RNAs which suffer alternate processing under cold stress conditions, however further analysis is necessary in order to confirm this finding for the *A. tequilana* genes.

Previous reports have described the synthesis and storage of fructans in the stems (head or piña) of agave plants. Wang and Nobel [6] also demonstrated the presence of low (3–5) DP fructans in the vascular tissue of mature leaves in *A. deserti* which correlated with the presence and activity of fructosyltransferase enzymes. The qRT-PCR results for *Atq1-SST-2* and *Atq6G-FFT-1* suggest a correlation between expression of the fructosyltransferase genes and carbohydrate storage organs since the highest levels of expression were found in stem tissues. On the other hand, although at lower levels, the presence of fructosyltransferase gene transcripts in leaf tissues of mature plants correlates with the fructosyltransferase activity and the synthesis of low DP fructans in the vascular tissue of mature leaves, observed for *A. deserti.* Low DP fructans could then be transported to the stem where higher DP fructans could be produced for storage.

The pattern of expression of the fructosyltransferase genes observed in Agave are similar to that found in *L. perenne* [9] where expression of a 1-SST gene was found to be higher in sheaths and enclosed tissue at the basal stem region than in mature leaf blades and emerged leaves. The 6G-FFT gene analyzed in *L. perenne* however, was also expressed to some extent in mature leaf blades and emerged leaves in addition to basal tissues.

Differences were observed for both fructosyltransferase genes between the vegetative stages (1 and 3 years) and mature plants (5 and 7 years). In 7 year-old plants higher levels of expression were observed in BL and ML tissue possibly indicating that an increase in low DP fructan synthesis is occurring as the plants begin to senescence. Further detailed analysis of transcript abundance, enzyme activity and fructan profiles throughout the life cycle of individual agave plants is necessary in order to determine the relationship between these factors and whether the agave fructosyltransferase genes are also regulated post-transcriptionally as in the case of *L. perenne* [9].

The invertase gene expression patterns also showed differences between the vegetative-state plants and mature plants with significant increases in expression levels of both *AtqVinv-1* and *AtqCwinv-1* in mid-leaf tissue. This expression pattern probably reflects the remobilization of carbohydrates that occurs as the plant becomes less photosynthetically active and enters into the stage of senescence. Extracellular invertases have been shown to be associated with delayed senescence [46] and it has been suggested that sugars may play a vital role in integrating environmental signals during leaf senescence, implying that enzymes involved in sucrose metabolism must also be involved [47].

The identification of *A. tequilana* transcripts for both FTs and invertases in anther, ovary and inflorescence cDNA libraries [36] suggests that sucrose and/or fructan metabolism may play roles such as signaling, osmoregulation or protection from stress. Several reports have shown that invertases are involved in the regulation of developmental processes in plants and in response to environmental cues or stress [48]–[50] and a recent report in *A. thaliana* described a transcription factor which regulates flowering in *A. thaliana* by modifying sucrose metabolism [51]. The environmental and internal signals that trigger the vegetative to inflorescence transition in Agave are unknown and day length and temperature do not seem to be important factors, therefore the potential involvement of carbohydrate accumulation and/or metabolism in this process should be addressed in more detail.

## Materials and Methods

### Ethics Statement

*A. tequilana* Weber var. Azul is the commercial agave cultivar used to produce tequila and is not an endangered agave species. All necessary permits were obtained for the described field studies and permission to sample plants from commercial plantations was obtained from Tequila Don Nacho S. A. de C. V. Agave producers in Jalisco State, Mexico and owners of the plantations. The manager of the production plant José de Jesus Trujillo (Chemical Engineer) was present during the sampling process.

### Plant Material

Tissues from Agave plants for qRT-PCR were collected from plantations of 1, 3, 5 and 7 year old plants located in the region of Los Altos de Jalisco, Mexico. Plants of 1 and 3 years were in the vegetative growth stage whereas plants of 5 and 7 years had already reached physiological maturity and the inflorescence removed by the farmers. Tissues from stem (S), leaf base (BL) and mid-leaf (ML) were dissected, frozen in liquid nitrogen and ground to a fine powder, then stored at –80°C until used.

### Bioinformatic Analysis

The *A. tequilana* transcriptome database was produced by preparing cDNA libraries from RNA obtained from different tissues of *A. tequilana* plants including roots, leaves, stems, flowers, meristems and bulbils (database access can be arranged by contacting J. Simpson: jsimpson@ira.cinvestav.mx or A. Martínez: aidamh@colpos.mx). cDNAs were inserted in the vector pDONR222 and 5′ sequenced by the Sanger method [52]. From 700–1500 bp of each cloned insert was sequenced and over 29,000 clones were analyzed (Martínez-Hernández et al. in preparation). BLAST searches for fructosyltransferases, fructan exohydrolases and invertase sequences were carried out on the database by using keywords and/or amino acid and cDNA sequences for the relevant genes from closely related species (*A. officinalis*, *T. aestivum* and *A. cepa*). Significant hits with a threshold e-value of 10^−6^ were selected. Open reading frames (ORFs) were translated using DNAstar software (EditSeq program) and multiple sequence alignments were performed with Clustal X software [53]. Comparison of sequences was carried out using the Neighbor-Joining method with 1000 bootstrap replicates [54] implemented in the MEGA 4 program [55]. The nucleotide and protein sequences reported in this manuscript have been submitted to GenBank under accession numbers JN790053-JN790064.

### Genomic and EST Sequence Analysis

In order to identify full-length cDNA clones for each gene of interest, plasmids carrying the longest sequenced inserts were subjected to restriction digestion. Inserts longer than the sequenced fragment were re-sequenced in both directions and by this method at least one full-length clone was identified for each distinct gene. Plasmids carrying selected cDNA fragments harboring Agave fructosyltransferase and invertase sequences were isolated by alkaline lysis [56] and when necessary complete inserts were sequenced using internal primers.

To determine the genomic sequence and exon/intron structure of each selected cDNA an overlapping PCR strategy was carried out using genomic DNA as a template and based on primers located within the ORF. PCR reactions were carried out with either recombinant Taq DNA Polymerase (Fermentas) or Phire Hot Start DNA Polymerase (FINNZYMES). PCR conditions varied in accordance with specific fragment and primer combinations. Amplified fragments were cloned in the TOPO 2.1 vector (Invitrogen) and sequenced by the Sanger method [52]. Homologous sequences were aligned to allow construction of the full-length genomic sequence. The number, length and position of exons and introns were obtained by comparison against the respective cDNA sequences.

### Expression Analysis

Total RNA was isolated from stem and leaf tissues of three distinct 1, 3, 5 and 7 year old *A. tequilana* Weber var. *azul* plants using the Trizol reagent (Invitrogen) and the PureLink Micro-to-Midi Total RNA Purification System (Invitrogen) according to the manufacturer’s instructions. cDNA templates for qRT-PCR amplification were prepared from pooled RNA’s from the three individual plants of each age by using specific primers (Table S3) and SuperScript™ III reverse transcriptase (Invitrogen) according to the manufacturer’s instructions. Each reaction contained 250 ng cDNA template obtained from ∼30 µg total RNA, 1× SYBR Green PCR Master Mix (Applied Biosystems) and 500 nM forward and reverse primers. Real-time PCR was performed in an ABI PRISM 7500 sequence detection system (Applied Biosystems) under the following thermal cycling conditions: 10 min at 95°C followed by a total of 40 cycles of 30 s at 95°C, 30 s at 60°C and 40 s at 72°C. Relative transcript abundance was calculated and normalized with respect to ubiquitin (UBQ11) to minimize variation in cDNA template levels. Data shown represent mean values obtained from at least three independent amplification reactions, and the error bars indicate the ±SE of the mean. All calculations and analyses were performed using 7500 Software v2.0.1 (Applied biosystems) and the 2^−ΔΔCt^ method with a relative quantification (RQ) confidence set at 95% [57]. Amplification efficiency (97.47% to 100.02%) for the primer sets was determined by amplification of a cDNA dilution series (1∶5). The specificity of the RT-PCR products was determined by a melting curve analysis with continual fluorescence data acquisition during the 65–95°C melt.

### Heterologous Expression in *Pichia pastoris*

Fructosyltransferase and Invertase enzymes are translated as preproteins containing a signal peptide. In order to identify the signal peptide sequence, full-length cDNAs were analyzed with the Signal P 3.0 software (http://www.cbs.dtu.dk/services/SignalP/) and by alignment of the amino acid sequences of the deduced Agave proteins with previously characterized fructosyltransferases. Based on these analyses, specific primers containing Eco RI and Xba I sites were designed to specifically amplify the mature protein-coding region (Table S3), using the cDNAs previously cloned in pDONR222 as template. PCR conditions were: 1 cycle of 94°C for 5 min, 30 cycles of 94°C for 30 s, 55°C for 30 s and 72°C for 2 min, followed by 1 cycle of 72°C for 5 min with Taq DNA polymerase (Invitrogen). The PCR products were cloned into the TOPO 2.1 vector (Invitrogen) and sequenced to verify the fidelity of the cloned fragment. Fragments with confirmed sequences were digested with Eco RI (Invitrogen) and Xba I (NEB) restriction enzymes and ligated into the pPICZαA expression vector (Invitrogen). The X-33 *P. pastoris* strain was transformed by electroporation with 10 µg of pPICZαA:ST2 (1-SST insert), pPICZαA:6G1 (6G-FFT insert), pPICZαA:Inv (Invertase insert) or with pPICZαA (empty vector) linearized with Pme I (Fermentas). Positive clones were selected on YPDS plus zeocin (100 mg/ml). Resistant colonies were also screened by PCR with vector specific primers for 5′ AOX1, 3′ AOX and α-factor (Invitrogen) and gene specific primers.

In order to produce the recombinant protein, single colonies were inoculated into 100 ml BMGY (1% yeast extract; 2% peptone; 100 mM potassium phosphate pH 6.0; 1,34% yeast nitrogen base (YNB); 4×10^−5^% biotin; 1% of glycerol) preculture medium and incubated at 28–30°C with shaking at 250 rpm until an OD_600_ = 2–6 was reached. The cells were harvested by centrifugation and resuspended in 20 ml of BMMY induction medium (BMGY with 0.5% of methanol instead of glycerol) and grown for 4 days (96 h) under the conditions described above. Methanol was added every 24 h to a final concentration of 1.5% to maintain induction.

### Purification of Recombinant Proteins

Protein purification was carried out by affinity chromatography. The supernatant was recovered and loaded onto a 10 ml column (Polyprep, Bio-Rad) containing 1 ml of previously equilibrated (50 mM NaH_2_PO_4_, 300 mM NaCl pH 8.0) Ni-NTA affinity resin (Quiagen). The resin was washed twice with 20 mM imidazol and twice eluted with 500 µl of elution buffer containing 250 mM of imidazol. Samples were dialyzed and concentrated in a Vivaspin 30000 MWCO column (GE Healthcare) and resuspended in 50 mM sodium acetate buffer pH 5.0. Protein extracts from *P. pastoris* strains transformed with empty vector, were obtained by precipitation of supernatant using ammonium sulfate until 80% saturation, and dialyzed as described above. Proteins were visualized in a Coomassie stained 10% SDS gel and concentration was determined by the method of Bradford [58] with the Protein Assay reagent (Bio-Rad) using Bovine Serum Albumin (BSA) as standard.

### Enzymatic Assays and Carbohydrate Analyses

Reactions were standardized and carried out for each enzyme as follows: for AtqCwinv1 reactions were carried out in 50 mM sodium acetate buffer pH 4.3 with 100 mM of sucrose or 100 mM of 1-kestose, using 10 µg of affinity purified protein, whereas for fructosyltransferase reactions were carried out in 50 mM sodium acetate buffer pH 5.5 with 100 mM of sucrose for Atq1-SST2 and either 100 mM of sucrose, 50 mM of 1-kestose or both for Atq6G-FFT1 at 30°C, using 10 µg of affinity purified protein for Atq1-SST-2 or 20 µg of total protein extract for Atq6G-FFT-1. The reactions were terminated by boiling for 5 minutes.

Reaction products were first visualized by TLC in a mobile phase consisting of butanol: ethanol:water (5∶3:2) and stained with aniline [59] and by HPAEC-PAD using an ion chromatographer Dionex ICS-3000 with a CarboPac PA-100 (4 mm×50 mm) guard-column and a CarboPac-PA100 (4 mm×250 mm) column (Dionex Corp., California, USA). The protocol developed by Mellado-Mojica and López (in preparation) was used: samples were diluted (1∶200) with deionized water and filtered through a 0.45 µm nylon membrane before injection of 25 µL of the samples. Fructan separation was achieved using a gradient of sodium acetate in 0.15 M NaOH at a column temperature of 25°C. The potentials applied for detection by amperometric pulse E1 (400 ms), E2 (20 ms), E3 (20 ms), and E4 (60 ms) were +0.1, −2.0, +0.6, and −0.1 V, respectively. Glucose, fructose, sucrose and 1-kestose molecules were identified by comparison with commercial standards. Neokestose and higher DP fructan standards were provided by Dr. Mercedes G. López.

## Supporting Information

Figure S1 ***Agave tequilana*** **var.** ***azul*** **plant for tissue colection.** A) Plant structure, B) Head or “piña", C) Leaves S-Stem, BL- Basal leaf section and ML-middle leaf section.
(PDF)

Figure S2 Semiquantitative RT-PCR expression profile of fructosyltransferase genes *Atq1-SST-1*, *Atq1-SST-2*, *Atq6G-FFT-1* and *Atq6G-FFT-2*. S-stem, BL-base of leaf, ML-middle leaf.
(PDF)

Figure S3 **Structural comparisons of recombinant proteins from** ***Agave tequilana*** **with respect to crystallized 1-FEH from** ***Cichorium intybus*** **with and without a histidine tag.** **A)** Atq1-SST2, **B)** Atq6G-FFT1 and **C)** AtqCwinv-1.
(PDF)

Figure S4 **Alignment of two different EST sequences encoding Atq6G-FFT-1 with and without the 9-bp exon of the β-fructosidase motif.** The red box indicates the 9 bp exon position.
(PDF)

Table S1 **Accession numbers for FT and invertase sequences used in the comparative analysis.**
(PDF)

Table S2 **List of functionally characterized proteins included in** **Figure 2** **.**
(PDF)

Table S3 **Primers used for analysis of expression (qRT-PCR) and for construction of** ***P. pastoris*** **heterologous expression vectors.**
(PDF)

## References

[1] Curbelo YG, Lopez MG, Bocourt R (2009). Fructans in Agave fourcroydes, potentialities for its utilization in animal feeding.. Cuban Journal of Agricultural Science.

[2] Hendry GAF (1993). Evolutionary Origins and Natural Functions of Fructans - a Climatological, Biogeographic and Mechanistic Appraisal.. New Phytologist.

[3] López MG, Mancilla-Margalli NA, Mendoza-Díaz G (2003). Molecular structures of fructans from Agave tequilana Weber var. azul.. Journal of Agricultural and Food Chemistry.

[4] Arrizon J, Morel S, Gschaedler A, Monsan P (2010). Comparison of the water-soluble carbohydrate composition and fructan structures of Agave tequilana plants of different ages.. Food Chemistry.

[5] Kawakami A, Yoshida M (2002). Molecular characterization of sucrose:sucrose 1-fructosyltransferase and sucrose:fructan 6-fructosyltransferase associated with fructan accumulation in winter wheat during cold hardening.. Biosci Biotechnol Biochem.

[6] Wang N, Nobel PS (1998). Phloem Transport of Fructans in the Crassulacean Acid Metabolism Species Agave deserti.. Plant Physiology.

[7] Pilonsmits EAH, Ebskamp MJM, Paul MJ, Jeuken MJW, Weisbeek PJ (1995). Improved Performance of Transgenic Fructan-Accumulating Tobacco under Drought Stress.. Plant Physiology.

[8] Wang N, Nobel PS (1998). Phloem Transport of Fructans in the Crassulacean Acid Metabolism Species Agave deserti.. Plant Physiol.

[9] Lasseur B, Lothier J, Djoumad A, De Coninck B, Smeekens S (2006). Molecular and functional characterization of a cDNA encoding fructan:fructan 6G-fructosyltransferase (6G-FFT)/fructan:fructan 1-fructosyltransferase (1-FFT) from perennial ryegrass (Lolium perenne L.).. J Exp Bot.

[10] Vijn I, Smeekens S (1999). Fructan: more than a reserve carbohydrate?. Plant Physiol.

[11] Van den Ende W, Coopman M, Clerens S, Vergauwen R, Le Roy K (2011). Unexpected Presence of Graminan- and Levan-Type Fructans in the Evergreen Frost-Hardy Eudicot Pachysandra terminalis (Buxaceae): Purification, Cloning, and Functional Analysis of a 6-SST/6-SFT Enzyme.. Plant Physiology.

[12] Fujishima M, Sakai H, Ueno K, Takahashi N, Onodera S (2005). Purification and characterization of a fructosyltransferase from onion bulbs and its key role in the synthesis of fructo-oligosaccharides in vivo.. New Phytol.

[13] Shiomi N (1989). Properties of Fructosyltransferases Involved in the Synthesis of Fructan in Liliaceous Plants.. Journal of Plant Physiology.

[14] Mancilla-Margalli NA, López MG (2006). Water-soluble carbohydrates and fructan structure patterns from Agave and Dasylirion species.. Journal of Agricultural and Food Chemistry.

[15] Verhaest M, Ende WV, Roy KL, De Ranter CJ, Laere AV (2005). X-ray diffraction structure of a plant glycosyl hydrolase family 32 protein: fructan 1-exohydrolase IIa of Cichorium intybus.. Plant J.

[16] Verhaest M, Lammens W, Le Roy K, De Ranter CJ, Van Laere A (2007). Insights into the fine architecture of the active site of chicory fructan 1-exohydrolase: 1-kestose as substrate vs sucrose as inhibitor.. New Phytol.

[17] Verhaest M, Van den Ende W, Yoshida M, Le Roy K, Peeraer Y (2004). Crystallization and preliminary X-ray diffraction study of fructan 1-exohydrolase IIa from Cichorium intybus.. Acta Crystallogr D Biol Crystallogr.

[18] Henson CA, Livingston DP, 3rd (1996). Purification and characterization of an oat fructan exohydrolase that preferentially hydrolyzes beta-2,6-fructans.. Plant Physiol.

[19] De Roover J, Vandenbranden, Van Laere A, Van den Ende W (2000). Drought induces fructan synthesis and 1-SST (sucrose:sucrose fructosyltransferase) in roots and leaves of chicory seedlings (Cichorium intybus L.).. Planta.

[20] Le Roy K, Verhaest M, Rabijns A, Clerens S, Van Laere A (2007). N-glycosylation affects substrate specificity of chicory fructan 1-exohydrolase: evidence for the presence of an inulin binding cleft.. New Phytol.

[21] Le Roy K, Lammens W, Verhaest M, De Coninck B, Rabijns A (2007). Unraveling the difference between invertases and fructan exohydrolases: a single amino acid (Asp-239) substitution transforms Arabidopsis cell wall invertase1 into a fructan 1-exohydrolase.. Plant Physiol.

[22] Yildiz S (2011). The Metabolism of Fructooligosaccharides and Fructooligosaccharide-Related Compounds in Plants.. Food Reviews International.

[23] Avila-Fernandez A, Olvera-Carranza C, Rudino-Pinera E, Cassab GI, Nieto-Sotelo J (2007). Molecular characterization of sucrose: sucrose 1-fructosyltransferase (1-SST) from Agave tequilana Weber var. azul.. Plant Science.

[24] Roitsch T, Balibrea ME, Hofmann M, Proels R, Sinha AK (2003). Extracellular invertase: key metabolic enzyme and PR protein.. Journal of Experimental Botany.

[25] Xiang L, Li Y, Rolland F, Van den Ende W (2011). Neutral invertase, hexokinase and mitochondrial ROS homeostasis: Emerging links between sugar metabolism, sugar signaling and ascorbate synthesis.. Plant Signal Behav.

[26] Sturm A (1999). Invertases. Primary structures, functions, and roles in plant development and sucrose partitioning.. Plant Physiology.

[27] Ritsema T, Verhaar A, Vijin I, Smeekens S (2004). Fructosyltransferase mutants specify a function for the beta-fructosidase motif of the sucrose-binding box in specifying the fructan type synthesized.. Plant Mol Biol.

[28] Bournay AS, Hedley PE, Maddison A, Waugh R, Machray GC (1996). Exon skipping induced by cold stress in a potato invertase gene transcript.. Nucleic Acids Research.

[29] Taliercio E, Scheffler J, Scheffler B (2010). Characterization of two cotton (Gossypium hirsutum L) invertase genes.. Mol Biol Rep.

[30] Shiomi N (2008). Food Biochemical Study on Fructans and Related Synthesis Enzymes.. J Applied Glycoscience.

[31] Wei JZ, Chatterton NJ (2001). Fructan biosynthesis and fructosyltransferase evolution: Expression of the 6-SFT (sucrose : fructan 6-fructosyltransferase) gene in crested wheatgrass (Agropyron cristatum).. Journal of Plant Physiology.

[32] Bocock PN, Morse AM, Dervinis C, Davis JM (2008). Evolution and diversity of invertase genes in Populus trichocarpa.. Planta.

[33] Ji X, Van den Ende W, Van Laere A, Cheng S, Bennett J (2005). Structure, evolution, and expression of the two invertase gene families of rice.. J Mol Evol.

[34] Ueno K, Onodera S, Kawakami A, Yoshida M, Shiomi N (2005). Molecular characterization and expression of a cDNA encoding fructan:fructan 6G-fructosyltransferase from asparagus (Asparagus officinalis).. New Phytol.

[35] Sherson SM, Alford HL, Forbes SM, Wallace G, Smith SM (2003). Roles of cell-wall invertases and monosaccharide transporters in the growth and development of Arabidopsis.. J Exp Bot.

[36] Simpson J, Hernandez AM, Juarez MJA, Sandoval SD, Villarreal AS (2011). Genomic resources and transcriptome mining in Agave tequilana.. Global Change Biology Bioenergy.

[37] Ji X, Shiran B, Wan J, Lewis DC, Jenkins CL (2010). Importance of pre-anthesis anther sink strength for maintenance of grain number during reproductive stage water stress in wheat.. Plant Cell Environ.

[38] Altenbach D, Nuesch E, Meyer AD, Boller T, Wiemken A (2004). The large subunit determines catalytic specificity of barley sucrose:fructan 6-fructosyltransferase and fescue sucrose:sucrose 1-fructosyltransferase.. FEBS Lett.

[39] Lasseur B, Schroeven L, Lammens W, Le Roy K, Spangenberg G (2009). Transforming a fructan:fructan 6G-fructosyltransferase from perennial ryegrass into a sucrose:sucrose 1-fructosyltransferase.. Plant Physiol.

[40] Schroeven L, Lammens W, Kawakami A, Yoshida M, Van Laere A (2009). Creating S-type characteristics in the F-type enzyme fructan:fructan 1-fructosyltransferase of Triticum aestivum L. J Exp Bot.

[41] Ritsema T, Hernandez L, Verhaar A, Altenbach D, Boller T (2006). Developing fructan-synthesizing capability in a plant invertase via mutations in the sucrose-binding box.. Plant J.

[42] Van den Ende W, Lammens W, Van Laere A, Schroeven L, Le Roy K (2009). Donor and Selector Substrate Specificity Among Glycoside Hydrolase Family 32 Enzymes.. FEBS Journal.

[43] De Coninck B, Le Roy K, Francis I, Clerens S, Vergauwen R (2005). Arabidopsis AtcwINV3 and 6 are not invertases but are fructan exohydrolases (FEHs) with different substrate specificities.. Plant Cell and Environment.

[44] Bennett J, Ji XM, Van den Ende W, Schroeven L, Clerens S (2007). The rice genome encodes two vacuolar invertases with fructan exohydrolase activity but lacks the related fructan biosynthesis genes of the Pooideae.. New Phytologist.

[45] Francki MG, Walker E, Forster JW, Spangenberg G, Appels R (2006). Fructosyltransferase and invertase genes evolved by gene duplication and rearrangements: rice, perennial ryegrass, and wheat gene families.. Genome.

[46] Balibrea Lara ME, Gonzalez Garcia MC, Fatima T, Ehness R, Lee TK (2004). Extracellular invertase is an essential component of cytokinin-mediated delay of senescence.. Plant Cell.

[47] Wingler A, Purdy S, MacLean JA, Pourtau N (2006). The role of sugars in integrating environmental signals during the regulation of leaf senescence.. J Exp Bot.

[48] Hayes MA, Feechan A, Dry IB (2010). Involvement of abscisic acid in the coordinated regulation of a stress-inducible hexose transporter (VvHT5) and a cell wall invertase in grapevine in response to biotrophic fungal infection.. Plant Physiol.

[49] Qi X, Wu Z, Li J, Mo X, Wu S (2007). AtCYT-INV1, a neutral invertase, is involved in osmotic stress-induced inhibition on lateral root growth in Arabidopsis.. Plant Mol Biol.

[50] Ji XM, Raveendran M, Oane R, Ismail A, Lafitte R (2005). Tissue-specific expression and drought responsiveness of cell-wall invertase genes of rice at flowering.. Plant Mol Biol.

[51] Park CM, Seo PJ, Ryu J, Kang SK (2011). Modulation of sugar metabolism by an INDETERMINATE DOMAIN transcription factor contributes to photoperiodic flowering in Arabidopsis.. Plant Journal.

[52] Sanger F, Nicklen S, Coulson AR (1977). DNA Sequencing with Chain-Terminating Inhibitors.. Proceedings of the National Academy of Sciences of the United States of America.

[53] Thompson JD, Chenna R, Sugawara H, Koike T, Lopez R (2003). Multiple sequence alignment with the Clustal series of programs.. Nucleic Acids Research.

[54] Felsenstein J (1986). Jackknife, Bootstrap and Other Resampling Methods in Regression-Analysis - Discussion.. Annals of Statistics.

[55] Kumar S, Tamura K, Dudley J, Nei M (2007). MEGA4: Molecular evolutionary genetics analysis (MEGA) software version 4.0.. Molecular Biology and Evolution.

[56] Sambrook J, Russell DW, Harbor ColdSpring (2001). Molecular cloning : a laboratory manual.. N.Y.: Cold Spring Harbor Laboratory Press.

[57] Livak KJ, Schmittgen TD (2001). Analysis of relative gene expression data using real-time quantitative PCR and the 2(T)(-Delta Delta C) method.. Methods.

[58] Bradford MM (1976). A rapid and sensitive method for the quantitation of microgram quantities of protein utilizing the principle of protein-dye binding.. Analytical Biochemistry.

[59] Reiffova K, Nemcova R (2006). Thin-layer chromatography analysis of fructooligosaccharides in biological samples.. J Chromatogr A.

