# Supplementary material for: Molecular and Functional Characterization of Novel Fructosyltransferases and Invertases from Agave tequilana
Source: PLoS One. 2012 Apr 30;7(4):e35878. doi: 10.1371/journal.pone.0035878 (PMC3340406; doi:10.1371/journal.pone.0035878)

Figure S1. *Agave tequilana* var. *azul* plant for tissue colection. A) Plant structure, B) Head or “piña”, C) Leaves S-Stem, BL- Basal leaf section and ML-middle leaf section

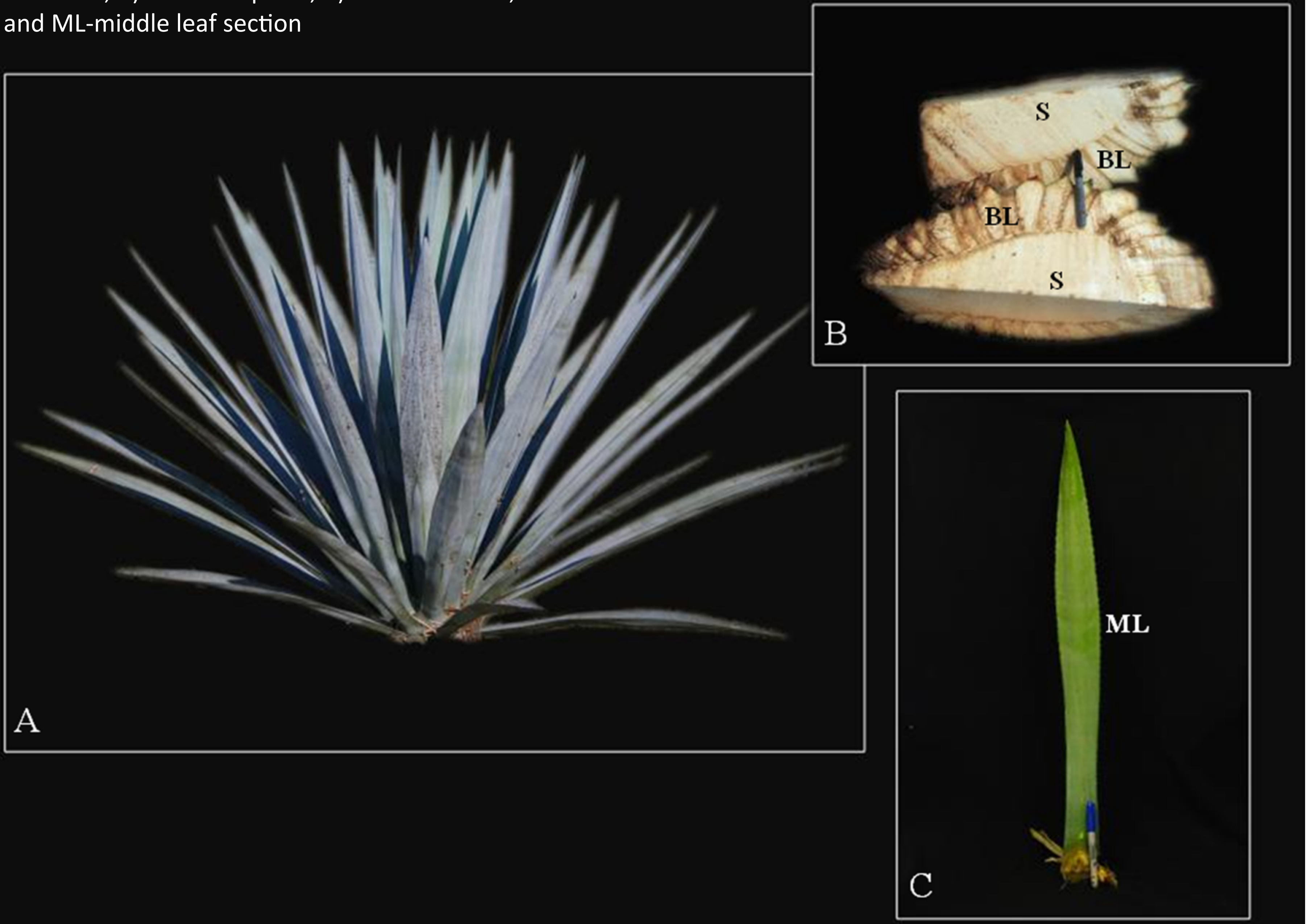

Supplement: Figure S1 — Agave tequilana var. azul plant for tissue colection. A) Plant structure, B) Head or “piña", C) Leaves S-Stem, BL- Basal leaf section and ML-middle leaf section. (PDF) [file pone.0035878.s001.pdf]
