# Supplementary material for: Molecular and Functional Characterization of Novel Fructosyltransferases and Invertases from Agave tequilana
Source: PLoS One. 2012 Apr 30;7(4):e35878. doi: 10.1371/journal.pone.0035878 (PMC3340406; doi:10.1371/journal.pone.0035878)

Figure S2. Semiquantitative RT-PCR expression profile of fructosyltransferases genes *Atq1-SST-1*, *Atq1-SST-2*, *Atq6G-FFT-1* and *Atq6G-FFT-2*. S-stem, BL-base of leaf, ML-middle leaf

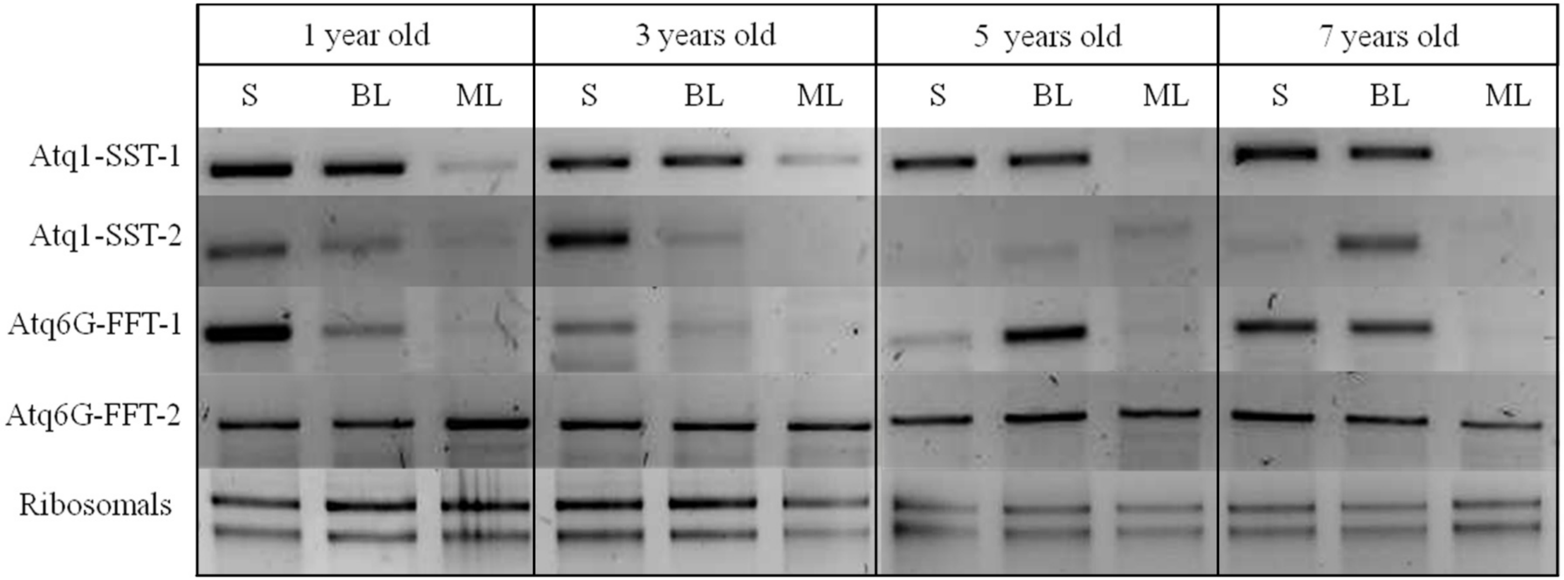

Supplement: Figure S2 — Semiquantitative RT-PCR expression profile of fructosyltransferase genes Atq1-SST-1, Atq1-SST-2, Atq6G-FFT-1 and Atq6G-FFT-2. S-stem, BL-base of leaf, ML-middle leaf. (PDF) [file pone.0035878.s002.pdf]
