# Supplementary material for: Molecular and Functional Characterization of Novel Fructosyltransferases and Invertases from Agave tequilana
Source: PLoS One. 2012 Apr 30;7(4):e35878. doi: 10.1371/journal.pone.0035878 (PMC3340406; doi:10.1371/journal.pone.0035878)
Supplement: Figure S3 — Structural comparisons of recombinant proteins from Agave tequilana with respect to crystallized 1-FEH from Cichorium intybus with and without a histidine tag. A) Atq1-SST2, B) Atq6G-FFT1 and C) AtqCwinv-1. (PDF) [file pone.0035878.s003.pdf]

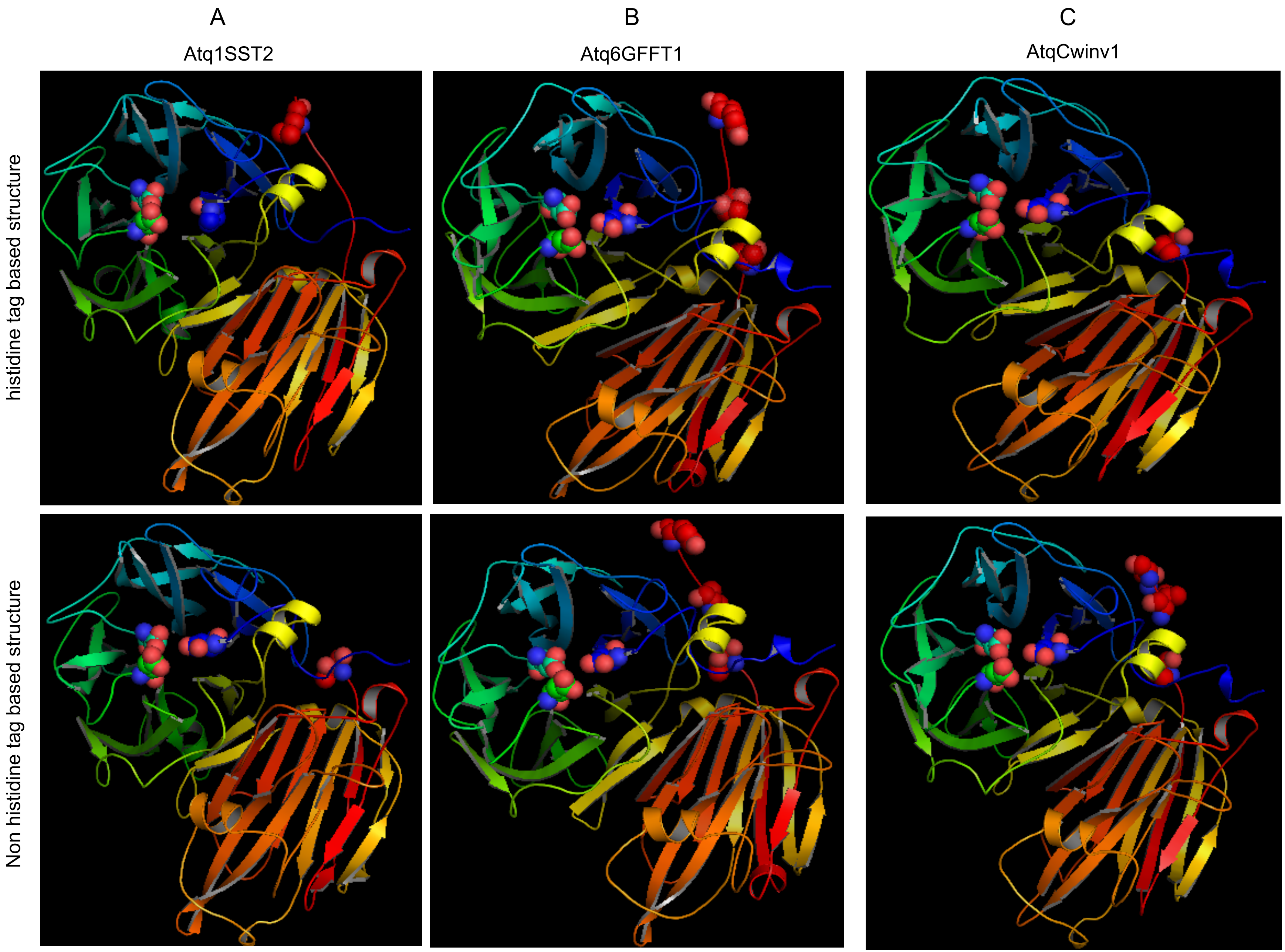

Figure S3. Structural comparisons of recombinant proteins from *Agave tequilana* with respect to crystallized 1-FEH from *Cichorium intybus* with and without a histidine tag. **A)** Atq1-SST2, **B)** Atq6G-FFT1 and **C)** AtqCwinv-1.
