# Supplementary material for: Molecular and Functional Characterization of Novel Fructosyltransferases and Invertases from Agave tequilana
Source: PLoS One. 2012 Apr 30;7(4):e35878. doi: 10.1371/journal.pone.0035878 (PMC3340406; doi:10.1371/journal.pone.0035878)
Supplement: Figure S4 — Alignment of two different EST sequences encoding Atq6G-FFT-1 with and without the 9-bp exon of the β-fructosidase motif. The red box indicates the 9 bp exon position. (PDF) [file pone.0035878.s004.pdf]

```
Atq6G-FFT1_p19 CTCAATCAAAACGTCACTGCCAACAAGAAGAAGAAAAACACAGCGTCTGGCGGTAGTCTTACCCATGGGCTCACCGGACCTCTACAATCCCATCGCTGGATCCCGTCCTCCGCTGAGAACTTTAA 125
Atq6G-FFT1_d08       CAAAACGTCACTGCCAACAAGAAGAAGAAAAACACAGCGTCTGGCGGTAGTCTTACCCATGGGCTCACCGGACCTCTACAATCCCATCGCTGGATCCCGTCCTCCGCTGAGAACTTTAA 119

Atq6G-FFT1_p19 GGTTACTCTCCCTCGCGCTGTCCGCTGCCCTTCTCTTGGCTCTGGTAGCCGTTACCTCGTTTCTCAACGTCAAGTCGGGTTCGGGGCTGGACTCGGGTTCGGGTTCCGACGAGGACGAGTTCCCG 250
Atq6G-FFT1_d08 GGTTACTCTCCCTCGCGCTGTCCGCTGCCCTTCTCTTGGCTCTGGTAGCCGTTACCTCGTTTCTCAACGTCAAGTCGGGTTCGGGGCTGGACTCGGGTTCGGGTTCCGACGAGGACGAGTTCCCG 244

Atq6G-FFT1_p19 TGGACCAAAAGGATGCTGACGTGGCAGCGGGCCGGGTTCCATTTCAGAACCGTTAAGAACTATATGACCGATCCATGTGGTCCAATGTATCACAAGGGCTGGTACCATCTCTTCTACCAGCACAA 375
Atq6G-FFT1_d08 TGGACCAAAAGGATGCTGACGTGGCAGCGGGCCGGGTTCCATTTCAGAACCGTTAAGAACTATATGACCG         GTCCAATGTATCACAAGGGCTGGTACCATCTCTTCTACCAGCACAA 360

Atq6G-FFT1_p19 TCCGAACTATTCCTTCTGGGACTACACCATGTCCTGGGGTCATGCTGTGTCTCGCGACCTACTCAACTGGTACCACCTCCCCGTCGCCATCCAGCCGGACCACTGGTACGATGTCTGGGGCGACT 500
Atq6G-FFT1_d08 TCCGAACTATTCCTTCTGGGACTACACCATGTCCTGGGGTCATGCTGTGTCTCGCGACCTACTCAACTGGTACCACCTCCCCGTCGCCATCCAGCCGGACCACTGGTACGATGTCTGGGGCGACT 485

Atq6G-FFT1_p19 GGACGGGCTCCATCATGAGACAATCGGACGGCAGGATCGTACTGCTGTACACCGGCATCACCGGCCGCAAAGAAGCCAAGAGGCAGGTCATCAATGTCGCAACGGCTGACGATCCCTCCGATCCG 625
Atq6G-FFT1_d08 GGACGGGCTCCATCATGAGACAATCGGACGGCAGGATCGTACTGCTGTACACCGGCATCACCGGCCGCAAAGAAGCCAAGAGGCAGGTCATCAATGTCGCAACGGCTGACGATCCCTCCGATCCG 610

Atq6G-FFT1_p19 CTCCTACTCAGATGGTCCAAGTATGAGGGCAACCCGGTGCTGCTTCCGCCTCCGGGCATCGAGCGGGAGGATTTCAGAGACCCCAGTCCTTTCTGGTACAACTCGTCAGATTCCACATGGTACTT 750
Atq6G-FFT1_d08 CTCCTACTCAGATGGTCCAAGTATGAGGGCAACCCGGTGCTGCTTCCGCCTCCGGGCATCGAGCGGGAGGATTTCAGAGACCCCAGTCCTTTCTGGTACAACTCGTCAGATTCCACATGGTACTT 735

Atq6G-FFT1_p19 GGTGTTCGGCTCTAGGAACGAGTCATTGAACCACGCCGGGATTGCCCTTGTCTACACCACCAGGGACTTCATCTCCTTCAACCTCCTCCCACACTACTTGCACTCCGTCAAGGAGATCGGCATGT 875
Atq6G-FFT1_d08 GGTGTTCGGCTCTAGGAACGAGTCATTGAACCACGCCGGGATTGCCCTTGTCTACACCACCAGGGACTTCATCTCCTTCAACCTCCTCCCACACTACTTGCACTCCGTCAAGGAGATCGGCATGT 860

Atq6G-FFT1_p19 GGGAGTGCGTCGAGCTCTACCCCGTCGCCGCTGCAGGCCCCTCGGCCAACCGGGGACTCGACCCCTTTGTGATGCCCGGGGAGAACGTGAAGCACGTGCTGAAATCGAGCGTCAACGACGAATGG 1000
Atq6G-FFT1_d08 GGGAGTGCGTCGAGCTCTACCCCGTCGCCGCTGCAGGCCCCTCGGCCAACCGGGGACTCGACCCCTTTGTGATGCCCGGGGAGAACGTGAAGCACGTGCTGAAATCGAGCGTCAACGACGAATGG 985

Atq6G-FFT1_p19 CACGATTACTATGCGATCGGCACATTTGATACGGGCACGATGACATGGACCCCCGACGATGAGACGGTGGATGTGGGGATGGGCATGAGGTACGACTGGGGCAAGTTTTATGCGTCGAGAACGTT 1125
Atq6G-FFT1_d08 CACGATTACTATGCGATCGGCACATTTGATACGGGCACGATGACATGGACCCCCGACGATGAGACGGTGGATGTGGGGATGGGCATGAGGTACGACTGGGGCAAGTTTTATGCGTCGAGAACGTT 1110

Atq6G-FFT1_p19 TTACGACCAGATGAAGCAGAGGAGGATACTGTGGGGGTACGTCGGAGAGACTGACAGTCAAAATGCTGATATTCAAAAGGGTTGGGCCTCATTTCAGGCCCTTCCCCGAGAAGTGTTGTTCGACT 1250
Atq6G-FFT1_d08 TTACGACCAGATGAAGCAGAGGAGGATACTGTGGGGGTACGTCGGAGAGACTGACAGTCAAAATGCTGATATTCAAAAGGGTTGGGCCTCATTTCAGGCCCTTCCCCGAGAAGTGTTGTTCGACT 1235

Atq6G-FFT1_p19 TGAAAACAGGCAGCAACCTTCTCACTTGGCCGGTCGAGGAGGTGAAGGGGCTTAGAATGAGAAGCAGAAATTTCAGTAACATCGTCGTCGAAAAGGGATCCACAGTCGAGCTTGATATTGGGGAT 1375
Atq6G-FFT1_d08 TGAAAACAGGCAGCAACCTTCTCACTTGGCCGGTCGAGGAGGTGAAGGGGCTTAGAATGAGAAGCAGAAATTTCAGTAACATCGTCGTCGAAAAGGGATCCACAGTCGAGCTTGATATTGGGGAT 1360

Atq6G-FFT1_p19 GCCAACCAGTTGGACATAGAAGTCGAGTTTGAGATCAACAAAGAGGACCTCGAGGCTGCGACCGTGGCTGATGTCGCCTACAACTGCACCAGCGGCAGTGCAGCCGCCCGAGGCCCCCTCGGACC 1500
Atq6G-FFT1_d08 GCCAACCAGTTGGACATAGAAGTCGAGTTTGAGATCAACAAAGAGGACCTCGAGGCTGCGACCGTGGCTGATGTCGCCTACAACTGCACCAGCGGCAGTGCAGCCGCCCGAGGCCCCCTCGGACC 1485

Atq6G-FFT1_p19 GTTTGGGCTACTCGTTCTCGCGAACAGGGATCTCACCGAGCAGACGGCAACATACTTTTACGTTAGCAGGGAAGCTGATGGCAGCGTTCGGACTCACTTTTGCCAGGATGAATTGAGATCAACCA 1625
Atq6G-FFT1_d08 GTTTGGGCTACTCGTTCTCGCGAACAGGGATCTCACCGAGCAGACGGCAACATACTTTTACGTTAGCAGGGAAGCTGATGGCAGCGTTCGGACTCACTTTTGCCAGGATGAATTGAGATCAACCA 1610

Atq6G-FFT1_p19 AGGCCAAAAATATCGTTAAGAGGGTGGTAGGGAACACATTTCCAGTGCTGACTGGTGAAACGTTGTCCGTGAGAACACTGGTGGATCATTCCATTGTGGAAAGCTTCGCTCAAGGAGGCCGGACG 1750
Atq6G-FFT1_d08 AGGCCAAAAATATCGTTAAGAGGGTGGTAGGGAACACATTTCCAGTGCTGACTGGTGAAACGTTGTCCGTGAGAACACTGGTGGATCATTCCATTGTGGAAAGCTTCGCTCAAGGAGGCCGGACG 1735

Atq6G-FFT1_p19 AGCACCACATCTCGCGCGTATCCAACAGAGGCAATATACGAGGATGCGCGAGTCTTCCTCTTCAACAACGCAACCGGCGCTACCGTAACGGCCAAGAGTGTGAAGATATGGCACATGAACTCCCC 1875
Atq6G-FFT1_d08 AGCACCACATCTCGCGCGTATCCAACAGAGGCAATATACGAGGATGCGCGAGTCTTCCTCTTCAACAACGCAACCGGCGCTACCGTAACGGCCAAGAGTGTGAAGATATGGCACATGAACTCCCC 1860

Atq6G-FFT1_p19 CGTCAACGAGCTCTATCAGTTCCCAAGCTTCCAGGCTCACTCAGGAGACGATTTTCAGCTCCATATTSCCTGATGTTATCATTTATGATTGGCATATTAATTCCATTTGCTGTGTCTTCGTATTG 2000
Atq6G-FFT1_d08 CGTCAACGAGCTCTATCAGTTCCCAAGCTTCCAGGCTCACTCAGGAGACGATTTTCAGCTCCATATTSCCTGATGTTATCATTTATGATTGGCATATTAATTCCATTTGCTGTGTCTTCGTATTG 1985

Atq6G-FFT1_p19 TGTTGTGTTAGASCAATAATTATTAGTGTGTAATGCAAATAAGAATAGTATTATAATTATTCATCTATATAAAAGAGAGGTTTTCTCAAAAA                                  2092
Atq6G-FFT1_d08 TGTTGTGTTAGASCAATAATTATTAGTGTGTAATGCAAATAAGAATAGTATTATAATTATTCATCTATATAAAAGAGAGGTTTTYTCAAAAA                                  2077
```

Figure S4. Alignment of two different EST sequences encoding Atq6G-FFT-1 with and without the 9-bp exon of the β-fructosidase motif as indicated by red box.
