# Supplementary material for: Molecular and Functional Characterization of Novel Fructosyltransferases and Invertases from Agave tequilana
Source: PLoS One. 2012 Apr 30;7(4):e35878. doi: 10.1371/journal.pone.0035878 (PMC3340406; doi:10.1371/journal.pone.0035878)
Supplement: Table S1 — Accession numbers for FT and invertase sequences used in the comparative analysis. (PDF) [file pone.0035878.s005.pdf]

Table S1. Accession numbers for FT and invertase sequences used in the comparative analysis**.**

Class	No. Accession	Species	Code
Monocotyledon	AJ006066	Allium cepa	Ac1SST
Monocotyledon	Y07838	Allium cepa	Ac6GFFT
Monocotyledon	AF211253	Agropyron cristatum	Ac6SFT
Monocotyledon	AJ006067	Allium cepa	AcInv
Monocotyledon	AB084283	Asparagus officinalis	Ao6GFFT
Monocotyledon	AF002656	Asparagus officinalis	AoInv
Monocotyledon	AB244731	Asparagus officinalis	AoCwinv
Monocotyledon	AY098442	Allium sativum	As1SST
Monocotyledon	EU026119	Agave tequilana	1-FFT Agave
Monocotyledon	DQ535031	Agave tequilana	SSTAg
Monocotyledon	JN790053	Agave tequilana	Atq1SST-1
Monocotyledon	JN790054	Agave tequilana	Atq1SST-2
Monocotyledon	JN790055	Agave tequilana	Atq6GFFT-1
Monocotyledon	JN790056	Agave tequilana	Atq6GFFT-2
Monocotyledon	JN790057	Agave tequilana	AtqCwinv-1
Monocotyledon	JN790058	Agave tequilana	AtqVinv-1
Monocotyledon	GQ247882	Bromus pictus	Bp1FEH
Monocotyledon	FJ424612	Bromus pictus	Bp6SFT
Monocotyledon	AJ297369	Festuca arundinacea	Fa1SST
Monocotyledon	AJ605333	Hordeum vulgare	Hv1FEH
Monocotyledon	AJ567377	Hordeum vulgare	Hv1SST
Monocotyledon	X83233	Hordeum vulgare	Hv6SFT
Monocotyledon	AJ534447	Hordeum vulgare	HvCwinv
Monocotyledon	AJ623275	Hordeum vulgare	HvInv
Monocotyledon	DQ016297	Lolium perenne	Lp1FEH1
Monocotyledon	AY245431	Lolium perenne	Lp1SST
Monocotyledon	AF494041	Lolium perenne	Lp6FFT
Monocotyledon	AF492836	Lolium perenne	Lp6GFFT
Monocotyledon	CAD58681	Lolium temulentum	LtInv
Monocotyledon	AAO21213	Musa acuminata	MaCwinv
Monocotyledon	AY578158	Oryza sativa	OsCwinv1
Monocotyledon	AY578160	Oryza sativa	OsCwinv3
Monocotyledon	AAT84404	Oryza sativa	OsCwinv4
Monocotyledon	AY578162	Oryza sativa	OsCwinv5
Monocotyledon	AF276703	Oryza sativa	OsInv2
Monocotyledon	AF276704	Oryza sativa	OsInv3
Monocotyledon	AF192394	Poa secunda	Ps6SFT
Monocotyledon	AY302083	Saccharum hybrid cultivar Pindar	ShInv
Monocotyledon	AJ508387	Triticum aestivum	Ta1FEH
Monocotyledon	AB088409	Triticum aestivum	Ta1FFT1
Monocotyledon	AB088410	Triticum aestivum	Ta1FFT2

Monocotyledon	AB029888	Triticum aestivum	Ta1SST
Monocotyledon	AM075205	Triticum aestivum	Ta6FEH
Monocotyledon	AB029887	Triticum aestivum	Ta6SFT
Monocotyledon	AF030420	Triticum aestivum	TaCwinv
Monocotyledon	AF069309	Triticum aestivum	TaInv1
Monocotyledon	AJ635225	Triticum aestivum	TaInv2
Monocotyledon	AF050129	Zea mays	ZmCwinv1
Monocotyledon	AF050128	Zea mays	ZmCwinv2
Monocotyledon	AF043346	Zea mays	ZmCwinv3
Monocotyledon	AF043347	Zea mays	ZmCwinv4
Monocotyledon	U16123	Zea mays	ZmInv
Dicotyledon	X74514	Arabidopsis thaliana	AtCwinv1
Dicotyledon	AY039610	Arabidopsis thaliana	AtInv1
Dicotyledon	AF274298	Brassica oleracea	BoInv1
Dicotyledon	AJ508534	Beta vulgaris	Bv6FEH
Dicotyledon	AJ277455	Beta vulgaris	BvInv1
Dicotyledon	AJ242538	Cichorium intybus	Ci1FEHI
Dicotyledon	AY323935	Cichorium intybus	Ci1FEHIIa
Dicotyledon	U84398	Cichorium intybus	Ci1FFT
Dicotyledon	U81520	Cichorium intybus	Ci1SST
Dicotyledon	Y11124	Cichorium intybus	CiCwinv
Dicotyledon	AJ419971	Cichorium intybus	CiInv1
Dicotyledon	AJ509808	Campanula rapunculoides	Cr1FEH
Dicotyledon	AJ000481	Cynara scolymus	Cs1FFT
Dicotyledon	Y09662	Cynara scolymus	Cs1SST
Dicotyledon	X75352	Daucus carota	DcInv
Dicotyledon	AJ009756	Helianthus tuberosus	Ht1FFT
Dicotyledon	AJ009757	Helianthus tuberosus	Ht1SST
Dicotyledon	AAD01606	Ipomoea batatas	IbInv1
Dicotyledon	AF506006	Lycopersicon esculentum	LeCwinv
Dicotyledon	AJ305044	Nicotiana tabacum	NtInv
Dicotyledon	D11350	Solanum lycopersicum	SlInv
Dicotyledon	X97642	Tulipa gesneriana	TgInv
Dicotyledon	AJ250634	Taraxacum officinalis	To1SST
Dicotyledon	AM231149	Vernonia herbacea	Vh1FEH
