# Supplementary material for: Molecular and Functional Characterization of Novel Fructosyltransferases and Invertases from Agave tequilana
Source: PLoS One. 2012 Apr 30;7(4):e35878. doi: 10.1371/journal.pone.0035878 (PMC3340406; doi:10.1371/journal.pone.0035878)
Supplement: Table S2 — List of functionally characterized proteins included in Figure 2 . (PDF) [file pone.0035878.s006.pdf]

Table S2. List of functionally characterized proteins included in Figure 2.

Species	Code	Reference
Cynara scolymus	Cs1FFT	Febs Letters 427: 25-28
Cynara scolymus	Cs1SST	Proc Natl Acad Sci U S A 97: 8699-8704<br>Plant J 12: 1057-1065
Taraxacum officinalis	To1SST	Plant Physiol 123: 71-79
Allium cepa	Ac1SST	Plant Physiol 117: 1507-1513
Allium cepa	Ac6GFFT	Plant J 11: 387-398
Agropyron cristatum	Ac6SFT	J Plant Physiol: 1203-1213
Allium cepa	AcInv	Plant Physiol 117: 1507-1513
Asparagus officinalis	Ao6GFFT	New Phytol 165: 813-824
Arabidopsis thaliana	AtCwInv1	Plant Physiol 145: 616-625
Agave tequilana	Atq1SST	Plant Science 478–486
Bromus pictus	Bp1FEH	Planta 231: 13-25
Bromus pictus	Bp6SFT	J Plant Physiol 168: 493-499
Beta vulgaris	Bv6FEH	Plant Journal 36: 697-710
Cichorium intybus	Ci1FEHI	Plant Journal 24: 447-456
Campanula rapunculoides	Cr1FEH	Functional Plant Biol 34: 972-983
Festuca arundinacea	Fa1SST	Plant Physiol 124: 1217-1227
Helianthus tuberosus	Ht1FFT	Febs Letters 427: 25-28
Helianthus tuberosus	Ht1SST	Nat Biotechnol 16: 843-846
Hordeum vulgare	Hv1SST	New Phytol 161: 735-748
Hordeum vulgare	Hv6SFT	Proc Natl Acad Sci U S A 92: 11652-11656
Hordeum vulgare	HvInv	New Phytol 161: 735-748
Ipomoea batatas	IbInv1	J Agric Food Chem 51: 1494-1499
Lolium perenne	Lp1FEH1	J Exp Bot 58: 1969-1983
Lolium perenne	Lp1SST	J Plant Physiol 160: 1385-1391
Lolium perenne	Lp6GFFT	J Exp Bot 57: 2719-2734

Oryza sativa	OsInv2	New Phytol 173: 50-62
Oryza sativa	OsInv3	New Phytol 173: 50-62
Triticum aestivum	Ta1FEH	Plant J 36: 697-710
Triticum aestivum	Ta1FFT1	Planta 223: 90-104
Triticum aestivum	Ta1SST	Planta 223: 90-104
Triticum aestivum	Ta6FEH	J Exp Bot 57: 213-223
Triticum aestivum	Ta6SFT	Biosci Biotechnol Biochem 66: 2297-2305
Vernonia herbacea	Vh1FEH	Plant Cell Physiol 49: 1185-1195
