# Supplementary material for: Molecular and Functional Characterization of Novel Fructosyltransferases and Invertases from Agave tequilana
Source: PLoS One. 2012 Apr 30;7(4):e35878. doi: 10.1371/journal.pone.0035878 (PMC3340406; doi:10.1371/journal.pone.0035878)
Supplement: Table S3 — Primers used for analysis of expression (qRT-PCR) and for construction of P. pastoris heterologous expression vectors. (PDF) [file pone.0035878.s007.pdf]

Table S3 Primers used for analysis of expression (qRT-PCR) and for construction of *P. pastoris* heterologous expression vectors.

Gen	Primer	Sequence	Application
Atq1-SST-2	TRST2fwd	5’-GTAGCTCCATGATGAGAAGG-3’	Analysis of expression
Atq1-SST-2	TRST2rev	5’-AACGACTTTGAGCGACTTGG-3’	Analysis of expression
Atq6G-FFT-1	TR6G1fwd	5’-GAGGCAATATACGAGGATGC-3’	Analysis of expression
Atq6G-FFT-1	TR6G1rev	5’-ACTGATAGAGCTCGTTGACG-3’	Analysis of expression
AtqCwinv-1	TRInv1fwd	5’-AAGGCAGAACCTGCATCACG-3’	Analysis of expression
AtqCwinv-1	TRInv1rev	5’-AAGCCTTGAGCTCTGATACC-3’	Analysis of expression
AtqVinv-1	AtINV2	5’-GTTCTTCGACGAGCAGAAGC-3’	Analysis of expression
AtqVinv-1	AtCw2MR	5’-TGGCAATATCAGCACTCTCG-3’	Analysis of expression
Ubiquitin 11	AtqUBQ 11-F	5’-GACGGGCGCACCCTTGCGGATTAC-3’	Analysis of expression
Ubiquitin 11	AtqUBQ 11-R	5’-TCCTGGATCTTCGCCTTGACATTG-3’	Analysis of expression
Atq6G-FFT-1	6Gfwd	5’-CTGAATTCTCCGACGAGGACGAGTTC-3’	Heterologous Expression
Atq6G-FFT-1	6Grev	5’-GTTCTAGAATATGGAGCTGAAAATCG-3’	Heterologous Expression
Atq1-SST-2	PICST2fwd	5’-GCTGAATTCGGCGTTGCTTCGAG-3’	Heterologous Expression
Atq1-SST-2	PICST2rev	5’-TACTCTAGAGGAGCTACAAGGC-3’	Heterologous Expression
AtqCwinv-1	PICINVfwd	5’-CTGAATTCTATCAAGAACTCATG3’-	Heterologous Expression
AtqCwinv-1	PICINVrev	5’-GTTCTAGAATTCCGTTCATCAAAG-3’	Heterologous Expression
